# YY1 is a transcriptional activator of the mouse LINE-1 Tf subfamily

**DOI:** 10.1093/nar/gkae949

**Published:** 2024-10-26

**Authors:** Karabi Saha, Grace I Nielsen, Raj Nandani, Yizi Zhang, Lingqi Kong, Ping Ye, Wenfeng An

**Affiliations:** Department of Pharmaceutical Sciences, South Dakota State University, 1055 Campanile Ave, Brookings, SD 57007, USA; Department of Pharmaceutical Sciences, South Dakota State University, 1055 Campanile Ave, Brookings, SD 57007, USA; Department of Pharmaceutical Sciences, South Dakota State University, 1055 Campanile Ave, Brookings, SD 57007, USA; Department of Pharmaceutical Sciences, South Dakota State University, 1055 Campanile Ave, Brookings, SD 57007, USA; Department of Pharmaceutical Sciences, South Dakota State University, 1055 Campanile Ave, Brookings, SD 57007, USA; Department of Pharmaceutical Sciences, South Dakota State University, 1055 Campanile Ave, Brookings, SD 57007, USA; Department of Pharmaceutical Sciences, South Dakota State University, 1055 Campanile Ave, Brookings, SD 57007, USA

## Abstract

Long interspersed element type 1 (LINE-1, L1) is an active autonomous transposable element in human and mouse genomes. L1 transcription is controlled by an internal RNA polymerase II promoter in the 5′ untranslated region (5′UTR) of a full-length L1. It has been shown that transcription factor YY1 binds to a conserved sequence at the 5′ end of the human L1 5′UTR and primarily dictates where transcription initiates. Putative YY1-binding motifs have been predicted in the 5′UTRs of two distinct mouse L1 subfamilies, Tf and Gf. Using site-directed mutagenesis, *in vitro* binding and gene knockdown assays, we experimentally tested the role of YY1 in mouse L1 transcription. Our results indicate that Tf, but not Gf subfamily, harbors functional YY1-binding sites in 5′UTR monomers and YY1 functions as a transcriptional activator for the mouse Tf subfamily. Activation of Tf transcription by YY1 during early embryogenesis is also supported by a reanalysis of published zygotic knockdown data. Furthermore, YY1-binding motifs are solely responsible for the synergistic interaction between Tf monomers, consistent with a model wherein distant monomers act as enhancers for mouse L1 transcription. The abundance of YY1-binding sites in Tf elements also raise important implications for gene regulation across the genome.

## Graphical Abstract

**Graphical Abstract:**
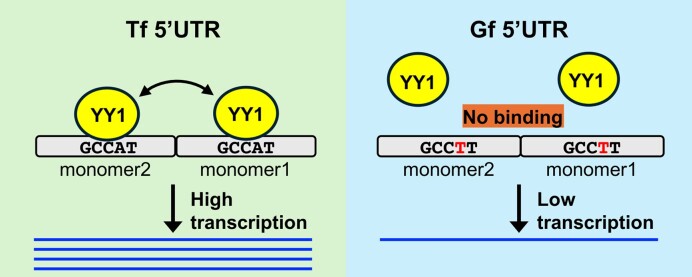

## Introduction

Transposable elements (TEs) constitute at least 46% of the human genome (1). The vast majority of human TEs are retrotransposons, which replicate in the genome through an RNA intermediate and are further divided into four classes: long terminal repeat (LTR) element, long interspersed element (LINE), short interspersed element (SINE) and the composite SINE-VNTR-Alu (SVA) element (1). LINE-1 (L1) is of particular interest as it is the only class of TEs that are both autonomous and active in the human genome (2–5). The vast majority of L1s in the human genome are 5′ truncated. A full-length L1 consists of a 5′ untranslated region (5′UTR), two tandem open reading frames (ORF1 and ORF2) that are separated by a short inter-orf spacer, and a 3′ untranslated region (3′UTR) (6). The transcription of a full-length L1 RNA is controlled by an internal Pol II promoter in the 5′UTR (7,8). L1 RNA has two essential functions during L1 retrotransposition: first, being translated into ORF1 and ORF2 proteins (9,10); second, being reverse transcribed by ORF2 protein into a new DNA copy (11,12). Thus, controlling L1 promoter activity represents a critical regulatory step for L1 replication cycle (13,14). One key aspect of L1 transcriptional regulation involves epigenetic mechanisms, such as DNA methylation, histone modification and, in germ cells, Piwi-interacting RNAs (15). Another important layer of L1 transcriptional regulation is the availability and binding of transcriptional factors to the L1 promoter region (14).

Transcription factor YY1 is a member of the C2H2 zinc finger protein family and is evolutionarily conserved among animals (16). In fact, human and mouse YY1 proteins are 98.6% identical to each other over the length of 414 amino acids (17,18). It is ubiquitously expressed in cell lines and human tissues (18–21). Since its initial discovery, YY1-binding sites have been found in promoters of many cellular and viral genes, activating or repressing them in a context dependent manner (22). Several models have been proposed to explain YY1’s seemingly divergent roles, including its recruitment of coactivators or corepressors (22). Recent studies indicate that YY1 is a structural regulator of enhancer-promoter interactions (21,23). It occupies active enhancers and promoter-proximal sequences across cell types (21,23). Through dimerization YY1 mediates interactions between enhancers and promoters at a genome-wide scale (21). Its association with these regulatory sequences is dependent on a known DNA-binding motif and augmented by YY1’s binding to RNA (21). Downregulation or ablation of YY1 leads to widespread changes in gene expression, with similar number of genes upregulated or downregulated (21,24), suggesting a model in which YY1 coordinates the positioning of other activators or repressors at individual promoters (21).

**Figure 1.**
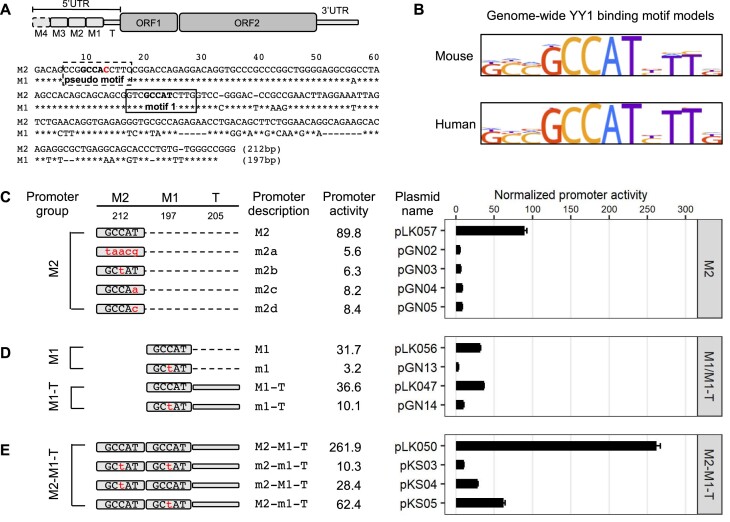
Effect of YY1 motif mutants on Tf_I promoter activity in F9 cells. (**A**) Alignment of Tf_I monomer 2 (M2) and monomer 1 (M1) consensus sequences. A schematic of mouse L1 containing four monomers is shown at the top (not to scale; T, tether). In the M1 sequence, nucleotide positions identical to M2 are marked by asterisks. Sequence gaps are represented by dashes. A previously predicted YY1-binding motif is located between nts 77–88 (solid box, termed ‘motif 1’). Toward the 5′ end of the monomers is another stretch of nucleotides highly similar to the consensus YY1-binding motif (dashed box, termed ‘pseudo motif’, which will be examined in later sections). (**B**) Mouse and human YY1-binding motif models from the HOCOMOCO database. (**C**) Normalized promoter activity of M2 constructs. Mutation to consensus YY1-binding motif 1 (GCCAT) is indicated by lowercases in red. (**D**) Normalized promoter activity of M1 and M1–Tether (M1–T) constructs. (**E**) Normalized promoter activity of M2–M1–T constructs. Mutation to one monomer at a time showed activity from the other monomer and tether. For panels (C–E), sequence organization of the promoters is illustrated on the left side. The length of M2, M1 and tether for each promoter is annotated (in base pairs). The dashed line represents domain(s) that were removed in reference to the two-monomer 5′UTR sequence (M2–M1–T). The x-axis indicates the normalized promoter activity, which is also listed under column ‘promoter activity’ for each promoter variant. The positive control construct, pCH117, had a normalized promoter activity of 355.0. Error bars represent standard errors of the mean (*n* = 4).

YY1 plays an important role in human L1 transcription. Human L1 5′UTR harbors a consensus YY1-binding site at nucleotide position (nt) 9–20 (25). The binding site has modest effect for the overall transcriptional output from the full-length 5′UTR (26) but is critical for the activity of the first 150 bp of L1 5′UTR (25,26). Moreover, an intact YY1-binding motif controls the transcription initiation from the 5′ end of the 5′UTR (26). However, whether YY1 regulates mouse L1 transcription has been inconclusive. Twenty-nine L1 subfamilies have amplified in the mouse genome since the split between mouse and rat about 13 million years ago (27). In the last one million years, at least four mouse L1 subfamilies (A_I, Tf_I, Tf_II and Gf_I) have been active, with the average age of elements within each subfamily varying from 0.21, 0.25, 0.27 to 0.75 million years, respectively (27). Like human L1, mouse L1 5′UTRs possess promoter activities (28–31). In contrast to human L1, mouse 5′UTRs are organized into tandemly repeated monomers, which are separated from ORF1 by a nonmonomeric tether sequence (27,32,33) (Figure 1A). Among the loci retaining at least a partial 5′UTR, A_I, Tf_I, Tf_II and Gf_I subfamilies average 3.7, 3.5, 3.1 and 2.3 monomers, respectively (34). Recently, we have shown that two-monomer consensus sequences from six L1 subfamilies differ in their promoter activities when tested in two separate murine cell lines in reporter assays (34). Putative YY1-binding motifs have been predicted in both Tf (30,31,35) and Gf monomers (36). A previous study attempted to determine the function of the putative YY1-binding site in Tf 5′UTRs by comparing the promoter activity of three genomic Tf promoters: one with a YY1 site and two without (37). Although a two-fold reduction in promoter activity was observed for the latter two Tf promoters these results are inconclusive due to the presence of other confounding mutations. Nevertheless, recent data showed that zygotic knockdown of YY1 reduced mouse L1 expression in developing embryos (38), implicating a regulatory role of YY1 on L1 transcription *in vivo*.

In this study we aim to determine the function of putative YY1-binding sites in the 5′UTR of mouse Tf_I and Gf_I subfamilies by performing site-directed mutagenesis, *in vitro* binding assays and small interfering RNA (siRNA) knockdown of YY1 protein in reporter assays. We report that the putative YY1-binding site is functional and required for Tf promoter while the predicted YY1-binding site in Gf_I promoter is not functional due to one nucleotide substitution in the core binding motif. In vitro binding assays show YY1’s interaction with the Tf promoter motif is inhibited by DNA methylation in a quantitative manner. Parallel reporter assays demonstrate that the human and mouse L1 5′UTRs respond to YY1 knockdown to similar degrees. Our reanalysis of published YY1 RNA-seq and ChIP-seq data supports an activator role of YY1 specifically for Tf_I/II subfamilies during early embryonic development.

## Materials and methods

### Plasmid construction

A detailed list of the promoter constructs, including the corresponding promoter sequences, is provided as a supplemental table (Supplementary Table S1). In all promoter constructs, the respective L1 promoter variant is positioned immediately upstream of the firefly luciferase (Fluc) reporter gene and flanked by two heterotypic SfiI sites (SfiI_L = GGCCAAAA/TGGCC and SfiI_R = GGCCTGTC/AGGCC; ‘/’ indicates the cleavage site). The double-SfiI cassette enables directional insert swapping via a single, robust restriction/ligation cycle (39). pCH117 is a positive control vector that contains the human L1RP 5′UTR as the ‘L1 promoter’ (34). pLK037 is a negative control vector that contains an empty double-SfiI cassette upstream of the Fluc reporter gene (34). Wild-type Tf_I promoter constructs (M2/pLK057, M1/pLK056, M1–T/pLK047 and M2–M1–T/pLK050) and wild-type Gf_I promoter constructs (M2/pLK063, M1/pLK062 and M2–M1–T/pLK051) have been described previously (34). Promoter variants containing nucleotide substitutions at the predicted YY1-binding motif were ordered as synthetic DNA fragments flanked by SfiI_L and Sfil_R restriction sites from either Genewiz (part of Azenta Life Sciences) or Twist Biosciences. pKS07 was derived from pCH117 by amplifying L1RP 5′UTR using a long forward primer extending from Sfil_L to the point mutation in YY1-binding motif and a reverse primer overlapping Sfil_R. Each synthetic DNA fragment was digested by SfiI (New England Biolabs) and ligated into SfiI digested backbone from pCH117 using T4 DNA ligase (New England Biolabs). All promoter variants were verified by Sanger sequencing (Elim Biopharmaceutics Inc). pMD5 is a *Sleeping Beauty* (SB) DNA transposon transfer vector encoding an m5’UTR-Fluc reporter transgene. pMD5 was constructed via a ligation of three DNA sequences: a 1647 bp NotI/NcoI fragment of pWA125 (40) containing the mouse L1 5′UTR, a 2262 bp NcoI/Acc65I fragment of pMD3 (41) containing the Fluc, SV40 polyA and SB 3′ inverted terminal repeat (ITR) and a 3183 bp Acc65I/NotI fragment of pT2BH (42) containing the plasmid backbone and SB 5′ITR. pMD5 was verified by whole plasmid sequencing (Plasmidsaurus Inc).

### Dual-luciferase promoter assay

Sublines of F9 mouse embryonal carcinoma cell line (ATCC CRL-1720), HeLa human cervical carcinoma cell line (ATCC CRL-2), NIH/3T3 mouse embryonic fibroblast cells (ATCC CRL-1658) were maintained in our lab. All three cell lines were propagated in a complete culture medium composed of Dulbecco’s modified Eagle’s medium/high glucose, 1% SG-200 and 10% fetal bovine serum (all from Cytiva Life Sciences). A reverse transfection protocol using Lipofectamine 3000 (Invitrogen) was followed (34). For F9 cells, a 96-well plate was coated with 0.1% gelatin for at least 30 min before adding transfection mix and cell suspension. Four replicate wells were allocated for each plasmid. In each well, 5 μl of transfection mix containing 10 ng of plasmid DNA was added followed by 100 μl of cell suspension (40 000 cells). The plate was incubated in a CO_2_ incubator at 37°C for 24 h before luminescence readout. For NIH/3T3 cells, 20 000 cells were added per well, and plates were incubated for 48 h. For HeLa cells, 24 000 cells were seeded per well, and plates were incubated for 48 h. Dual-Luciferase Reporter Assay System (Promega) was used to measure luciferase activities. Briefly, cells were lysed using 1× passive lysis buffer and transferred to a solid white flat-bottom 96-well plate (Greiner Bio-One). Fluc activity was read first on a GloMax Multi Detection System (Promega) followed by the measurement for Renilla luciferase (Rluc) activity. Signal integration time was set to one second per well. Mock transfected cells and empty wells were included to evaluate the assay background.

Promoter assays under siRNA knockdown of YY1 protein were performed in F9, NIH/3T3 and HeLa cells with modifications. A Yy1-specific siRNA or the negative control Allstars siRNA (QIAGEN) was co-transfected with an L1 promoter reporter plasmid using Lipofectamine 3000 (Invitrogen). The target sequences for human or mouse Yy1-specific siRNAs are provided (Supplementary Table S2). The siRNA transfection mix was prepared separately without P3000 and added to the 96-well plate after the addition of plasmid DNA transfection mix. A no siRNA control was included to evaluate the effect of RNA cotransfection on the promoter assay. Per well 5 pmol siRNA was used, equivalent to a concentration of 50 nM in 100 μl culture medium. After the addition of transfection mixes, 20 000 F9 cells, 10 000 NIH/3T3 cells or 14 000 HeLa cells were added to the well. Transfected F9 cells, NIH/3T3 cells and HeLa cells were incubated for 72 h, before luciferase measurements. Four replicate wells were allocated for each condition.

### Stable L1 promoter reporter cell lines

HCT116-h5'UTR-Fluc cell line, which reports the activity of human L1RP promoter, has been described previously (41). A similar approach was used to make a stable HCT116-m5'UTR-Fluc reporter cell line for mouse L1 promoter. Briefly, 90 000 wild-type HCT116 cells (ATCC CCL-247) were seeded in a 24-well plate and cotransfected with 100 ng of pMD5 and 20 ng of SB100X (42) using FuGENE HD reagent 24 h later. Cells were reseeded into 96-well plates at the density of 0.8 cells per well 24 h post-transfection. Single cell clones with Fluc signals were propagated in a complete culture medium composed of modified McCoy’s 5A medium, 1% SG-200, 100 unit/ml penicillin–streptomycin and 10% fetal bovine serum (all from Cytiva Life Sciences). For luciferase assays, 5 pmol control or human YY1-specific siRNAs and 10 000 HCT116-m5'UTR-Fluc (clone 1A7-C5) cells were added per well in a 96-well plate. At 72 h post-transfection, 20 μl of CellTiter Blue reagent (Promega) was added per well. Cells were incubated for 1 h before fluorescence was read on a GloMax Multi Detection System (Promega). Signal integration time was set to 1 s per well. Wells containing the complete culture medium were included to evaluate the assay background. Fluc activity was subsequently measured as described under ‘Dual-luciferase promoter assay’.

### Western blot

To verify siRNA knockdown efficiency, transfection was scaled up from a 96-well to 24-well plate by a factor of 6. siRNA transfection was set up in a similar way as the dual-luciferase assay in 96-well plate. Yy1-specific siRNAs were transfected separately as well as in combination. A total of 30 pmol siRNA was added per well. Allstars was used as a negative control. A total of 60 000 NIH/3T3 or HCT116 cells were added to each well. After 72 h of incubation, whole cell lysate was prepared using radioimmunoprecipitation assay (RIPA) buffer (Nalgene) in the presence of protease inhibitor cocktail (MilliporeSigma). Cell lysate was incubated on ice for 30 min, vortexed every 10 min and then centrifuged at 12 000 rpm for 20 min at 4°C. The supernatant was collected and measured for protein concentration using Pierce BCA Protein Assay Kit (Thermo Scientific). For western blot, a total of 15–20 μg whole cell lysate was resolved in a 10% or 12% TGX Stain-Free gel (Bio-Rad). At the end of the run, the gel was exposed to ultraviolet (UV) light for 2.5 min in Bio-Rad ChemiDoc XRS + System to activate the trihalo compound that is bound to tryptophan residues. Proteins were then transferred from gel into Immobilon-P PVDF membrane (MilliporeSigma). A fluorescent stain-free blot image was captured using Bio-Rad ChemiDoc XRS + System as the total protein signal for later normalization of protein loading. The membrane was blocked using Chemi Blot Blocking Buffer (Azure Biosystems) for 1 h at room temperature with gentle shaking. The membrane was subsequently cut into two parts. The upper portion was incubated with a mouse monoclonal antibody against YY1 (Santa Cruz Biotechnologies #sc-7341X) at 1:10 000 dilution and the lower portion was incubated with a mouse monoclonal antibody against histone H3 protein (Santa Cruz Biotechnologies #sc-517576) at 1:5000 dilution. After overnight incubation at 4°C with primary antibodies, the membrane was incubated with an Horseradish peroxidase-conjugated goat anti-mouse IgG secondary antibody (Bio-Rad #1706516) for 1 h at room temperature. Chemiluminescent signal was generated by adding ECL select western blot-detecting reagent (Cytiva Life Sciences) and imaged with Bio-Rad ChemiDoc XRS + System. Bio-Rad Image Labs v4.0 was used to quantify the YY1 protein signals and normalized to either total protein signals (e.g. from the fluorescent Stain-Free blot image) or the housekeeping H3 protein signals.

### Electrophoretic mobility shift assay

Details about all DNA fragments used in electrophoretic mobility shift assay (EMSA) are provided as a supplemental table (Supplementary Table S3). EMSA was performed using LightShift Chemiluminescent EMSA Kit (Thermo Scientific). Nuclear proteins were extracted using NE-PER Nuclear and Cytoplasmic Extraction Reagents (Thermo Scientific). In brief, F9 or NIH/3T3 cells at 90% confluence were trypsinized, centrifuged for 5 min at 500 g and washed with 1× phosphate-buffered saline. The pellet was resuspended in ice-cold cytoplasmic extraction reagent with protease inhibitor and centrifuged. The supernatant containing cytoplasmic proteins was removed. The pellet was resuspended in ice-cold nuclear extraction reagent with protease inhibitor, incubated on ice for a total of 40 min while vortexing for 15 s every 10 min and centrifuged for 10 min at 16 000 g at 4°C. The supernatant containing nuclear proteins was measured for protein concentration using Pierce BCA Protein Assay Kit (Thermo Scientific) and stored at −80°C until use. To generate biotin-labeled double-stranded DNA probe, a 5′ biotinylated 30-mer oligo and an unlabeled reverse complement oligo were annealed at a 1 μM concentration in 1× TE buffer in the presence of 50 mM NaCl. Unlabeled competitor DNA fragments were generated in the same fashion but at a concentration of 2 μM. Binding reactions were set up in a 20 μl volume containing 1× binding buffer, 2.5% glycerol, 5 mM MgCl_2_, 50 ng/ml poly dI dC, 0.05% NP-40, 20 fmol of annealed probe in the presence or absence of unlabeled competitor, and 4.5 μg of nuclear protein extract from either NIH/3T3 or F9 cells. Binding reactions were incubated for 20 min at room temperature without antibodies. After the binding reaction and when indicated, 2.5 μg of YY1 specific antibody (Santa Cruz Biotechnologies #sc-7341X) or mouse IgG isotype control (Thermo Fisher #02–6502) were added and incubated at room temperature for 20 more min. A 5% polyacrylamide gel was pre-run in 0.5× Tris-borate-EDTA (TBE) for 30 min. Each reaction was mixed with 5 μl of 5× loading dye, loaded and run on for 1.5 h at 100 V. Samples were then transferred to a nylon membrane (pre-soaked in cold 0.5× TBE for 10 min) at 380 mA for 30 min at 10°C. DNA was crosslinked to the membrane using Stratagene UV crosslinker 1800 instrument at 120 mJ/cm^2^ (using the auto-crosslink function). The membrane was blocked for 15 min using blocking buffer and incubated in conjugate/blocking buffer for 15 min. The membrane was washed four times for 5 min each, and equilibrated for 5 min in substrate equilibration buffer, and incubated in substrate working solution for 5 min. The chemiluminescence was captured by Bio-Rad ChemiDoc XRS + System.

### Differential expression analysis of RNA-seq data using TEtranscripts

RNA-seq data for mouse embryos (38,43) were downloaded from NCBI Gene Expression Omnibus (GEO) (Supplementary Table S4). Sample preparation is detailed in the original publications and summarized below. Briefly, Yy1-specific or control siRNAs were injected into the cytoplasm of zygotes at 2–3 h post-insemination (hpi). Eight-cell embryos and morulae were collected at 48 and 64 hpi, respectively. Each replicate contained 10 embryos. RNA-seq libraries were prepared using SMART-seq stranded kit (Takara) and sequenced in paired-end 53 bp for eight-cell embryos and in paired-end 150 bp for morulae. We conducted differential expression analysis of TE subfamilies using two different versions of TE annotation files (i.e. TE GTF/Gene Transfer Format files). To differentiate 29 mouse L1 subfamilies as defined by Sookdeo and colleagues (27), we used mm10 repeat library db20140131 (available from RepeatMasker website: http://www.repeatmasker.org). As a comparison and to reproduce results reported by Sakamoto and colleagues (38), we used the default TE annotation file GRCm38_Ensembl_rmsk_TE.gtf.gz (available at TEtranscripts website hosted by Dr Molly Hammell). Briefly, low-quality bases (Phred score < 20) and adapter sequences were trimmed from the 3′ end of the RNA-seq reads using Trim Galore. Sequencing reads were aligned to the mouse reference genome (GRCm38/mm10) using bowtie2 with parameters ‘–no-mixed –no-discordant –nofw –k 100 –p 24’. Resulting alignment files were fed into TEtranscripts (44) for differential expression analysis using parameters ‘–mode multi’. TE subfamilies belonging to LINE, SINE, LTR and DNA transposon classes were retained for downstream analyses. The difference between control and knockdown samples was considered statistically significant for a given TE subfamily when the adjusted *P* value (i.e. after multiple test correction) is <0.05 (Supplementary Table S5).

### Enrichment analysis of YY1 ChIP-seq data across TE subfamilies using T3E

Raw YY1 ChIP-seq data (45) were downloaded from NCBI GEO (Supplementary Table S4). The following ChIP-seq datasets were used for enrichment analysis: three replicates of untreated wild-type mESC J1 strain immunoprecipitated by YY1 antibody (Santa Cruz, sc-1703) and the corresponding input sample. All four libraries consisted of 75 bp paired-end reads from Illumina HiSeq4000 platform. Reads were preprocessed as previously described for T3E (46). Low-quality bases (Phred score < 20) and adapter sequences were trimmed from the 3′ end of the ChIP-seq reads. Sequencing reads were aligned to the mouse reference genome (GRCm38/mm10) using BWA-MEM v0.7.17 (47) with parameter ‘-a’, returning all mappings (both unimappers and multimappers). Duplicate reads, un-mapped reads and alignments to the mitochondrial chromosome and non-chromosomal scaffolds were removed. Resulting alignment files of three replicates and one input control were fed into T3E (46) for enrichment analysis. T3E repository was cloned from GitHub (https://github.com/michelleapaz/T3E) and installed on the institutional high-performance computing Linux cluster. Repeat annotations were derived from mm10 repeat library db20140131, available from the RepeatMasker website (http://www.repeatmasker.org), and filtered to retain only individual instances of TEs (i.e. LINE, SINE, LTR and DNA transposons). T3E calculates enrichment for TE subfamilies, not for individual TE copies, returning a fold-change (FC) and an empirical *P* value. For a given TE subfamily, *P* value < 0.01 is considered enrichment for the protein of interest. Out of 1159 TE subfamilies, 118 are enriched for YY1 binding (*P* < 0.01) across all three ChIP-seq samples. FC_mean is the mean FC among three replicates [the average is taken after converting each Log2 FC (log2FC) into FC] (Supplementary Table S6).

## Results

### Mutating the predicted YY1-binding site abolishes Tf_I promoter activity in reporter assays

In the 5′UTR of mouse L1 Tf subfamily, a YY1-binding motif was predicted at nt 77–88 (GTCGCCATCTTG) in the monomer consensus (31) (Figure 1A; motif 1). It contains the five core nucleotides (GCCAT) that are highly conserved among mouse and human YY1-binding sites (48) (Figure 1B). To evaluate the function of this putative YY1-binding site, we employed a single-vector dual-luciferase reporter assay in mouse F9 embryonal carcinoma cells as previously reported (34). In this assay, the Fluc is controlled by an L1 promoter variant. The Rluc is driven by herpes simplex virus thymidine kinase promoter and used to normalize transfection efficiency. To quantify the activity of an L1 promoter variant, Fluc/Rluc ratios are first calculated for each of the four replicate wells and then averaged. An average Fluc/Rluc ratio is similarly calculated for a negative control plasmid pLK037, which lacks a promoter sequence upstream of the Fluc (34), representing the assay background. The average Fluc/Rluc ratio of each promoter construct is subsequently normalized to that of pLK037 (i.e. setting the average Fluc/Rluc ratio of pLK037 to 1), giving rise to ‘normalized promoter activity’ (Figure 1C). Each experiment also includes a positive control plasmid pCH117, which contains a highly active human L1 promoter upstream of the Fluc coding sequence (34). The normalized promoter activity for pCH117 represents the assay dynamic range and is stated in the figure legend for each experiment.

In the first experiment, we compared the consensus M2 of Tf_I subfamily to four variants containing mutated YY1-binding sites (Figure 1C). The four mutant variants had either one or all five core nucleotides altered as compared with the core consensus YY1-binding sequence GCCAT. Three of the four variants had been previously shown to behave as a loss-of-function mutation in other promoter contexts: taacg (variant m2a; lowercase indicates substitution) (49), GCtAT (variant m2b) (50), GCCAa (variant m2c) (51). The fourth variant, GCCAc (m2d), was similar to variant m2c but had a T to C transition at the fifth nucleotide position instead. As expected, Tf_I M2 showed a normalized promoter activity of 89.8 (Figure 1C). In contrast, the four mutant promoter variants uniformly displayed minimal promoter activity, ranging from 5.6 to 8.4 and corresponding to 10.7 to 16.0-fold reduction as compared to the wild-type M2. The mutant promoters’ significant loss of activity suggests that the putative YY1-binding sequence is essential for transcriptional activation of Tf_I M2 when tested in isolation (i.e. when not linked to downstream 5′UTR sequence).

In the second experiment, we tested the function of the putative YY1-binding site in the context of M1 alone by introducing a single, centrally located nucleotide substitution as in m2b (Figure 1D). The mutant monomer 1 (m1) showed 10-fold less activity than the wild-type M1. Similarly, in the context of monomer 1 followed by the tether sequence (M1–T; T for tether), the mutant version (m1–T) had 3.6-fold reduced activity than the wild-type (Figure 1D). The higher residual activity seen in m1–T reflects the inherent contribution from the tether sequence (34).

In the third experiment, we conducted mutational analysis in the context of two-monomer Tf_I 5′UTR (M2–M1–T) (Figure 1E). As expected, the consensus M2–M1–T possessed significantly higher activity than M2 alone, M1 alone or M1–T (Figure 1C–E), reproducing the synergistic interaction among M2, M1 and T seen earlier (34). When the putative YY1 motif was mutated in both monomers (m2–m1–T), the promoter activity was reduced to 10.3, equivalent to that of m1–T. This observation suggests that the synergy among M2, M1 and T stems predominantly from the presence of the putative YY1 motifs in M2 and M1. Indeed, a singular mutation in M2 (m2-M1–T) reduced the activity to that of M1–T (28.4 versus 36.6, respectively). A singular mutation in M1 (M2–m1–T; Figure 1E) reduced its activity to a level that was like M2 alone (Figure 1C) (62.4 versus 89.8, respectively).

To exclude cell-specific artifacts, we repeated the experiments in the NIH/3T3 mouse embryonic fibroblast cell line and observed similar results (Supplementary Figure S1). Briefly, the four mutant M2 promoter variants uniformly displayed minimal activity, corresponding to 16- to 22-fold reduction relative to the wild-type (Supplementary Figure S1A). Variant m1 and m1–T showed 11-fold and 2.4-fold reduction in activity relative to their wild-type counterparts (Supplementary Figure S1B). When either or both YY1-binding motifs were mutated in the context of M2–M1–T promoter constructs a synergistic interaction between M2 and M1 was also reproduced (Supplementary Figure S1C). Taken together, these reporter assays suggest that the previously predicted YY1-binding motif in the consensus Tf_I monomer sequences is not only critical for each monomer’s own promoter activity but also responsible for the synergistic interaction between monomers.

### The putative YY1-binding motif in Tf_I 5′UTR interacts with YY1 protein

**Figure 2.**
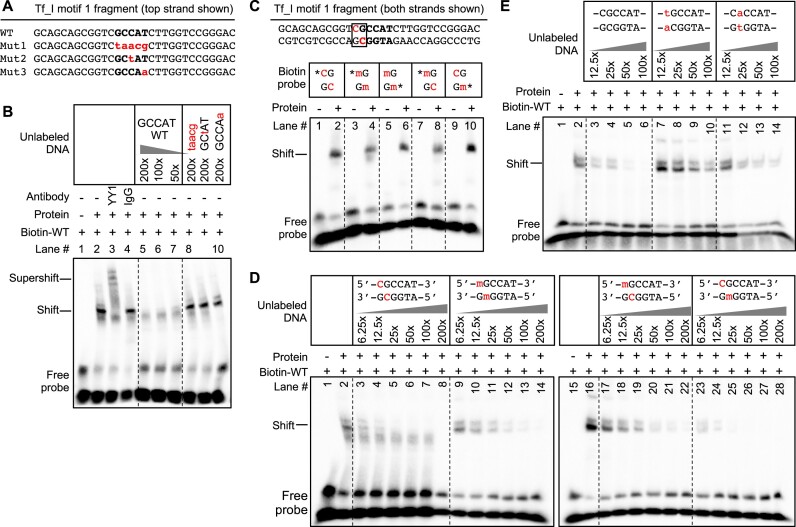
Interaction of YY1 protein with motif 1 is weakened by cytosine methylation in a strand-specific manner. (**A**) Wild-type and mutant DNA fragments used in EMSA. Each fragment was formed by annealing a sense stranded oligo (shown) with the corresponding antisense oligo (not shown). Mutations in the core binding motif are indicated by lowercases in red. (**B**) EMSA with Tf_I motif 1 fragments. The presence or absence of a biotin-labeled WT probe, antibody and nuclear protein extract from F9 cells is indicated by ‘+’ or ‘−’ symbols. Lane 3 had YY1-specific antibody and lane 4 had mouse IgG as a control. Lanes 5–10 had unlabeled DNA fragments as competitors in molar excess as indicated. (**C**) EMSA using unmethylated and variably methylated Tf_I motif 1 DNA fragments as probes. The complete sequence of the unmethylated fragment is shown at the top with the CpG dinucleotide boxed. Probes used for specific lanes are indicated by the CpG position (m, 5-methylcytosine; *, the 5′ biotin label). (**D**) EMSA using unmethylated and variably methylated Tf_I motif 1 fragments as competitor. A biotin-labeled unmethylated WT probe is used. Competitors used for specific lanes are indicated by the central motif (m, 5-methylcytosine). To facilitate comparison, reactions were run on two gels, processed and imaged simultaneously. (**E**) EMSA using mutant fragments at CpG position as competitor. A biotin-labeled unmethylated WT probe is used. The presence or absence of nuclear protein extract from F9 cells is indicated by ‘+’ or ‘−’ symbols for all panels.

To determine whether the putative YY1-binding motif in Tf_I 5′UTR interacts with YY1 protein, we utilized an EMSA. As a probe for motif 1, we used a biotin-labeled 30 bp double-stranded DNA fragment from Tf_I M2, with motif 1 centrally located (Figure 2A; WT fragment). Incubation with nuclear protein extract from F9 cells resulted in a shift in its migration (Figure 2B, lane 2). A supershift was observed with the addition of YY1-specific antibody (Figure 2B, lane 3) but not with the addition of non-specific mouse IgG (Figure 2B, lane 4), suggesting the interaction is mediated by YY1 protein. The shift was diminished by increasing amount of unlabeled DNA of the same sequence (Figure 2B, lanes 5–7) but not by three unlabeled DNA fragments (Mut1, Mut2 and Mut3 fragments shown in Figure 2A in which the core nucleotides were variably mutated as in the reporter assays) (Figure 2B, lanes 8–10). Similar results were obtained when nuclear protein extract from NIH/3T3 cells was used (Supplementary Figure S2B). The inability of these mutant DNA fragments to compete for YY1 binding highlights the presence of sequence-specific interaction of the predicted binding motif with YY1 protein.

YY1 shows preference of binding to unmethylated motifs (52–54). To test the effect of DNA methylation on YY1 binding to Tf_I motif 1, we assembled DNA probes containing 5-methylcytosine at the CpG position either in both strands (lanes 3–6; note lanes 3 and 4 and 5 and 6 differ only in which strand is biotin-labeled), only in the top strand (lanes 7 and 8), or only in the bottom strand (lanes 9 and 10) (Figure 2C). To our surprise, all isoforms showed a clear shift in the presence of YY1-containing nuclear protein extract from F9 cells. Although this binding assay was not quantitative, the probe with unmethylated top strand displayed a seemingly stronger signal (Figure 2C; lanes 9 and 10). To compare YY1 binding by unmethylated, symmetrically or asymmetrically methylated motifs, we used unlabeled isoforms ranging from 6.25-fold to 200-fold in excess to compete with the unmethylated probe (Figure 2D). In this semi-quantitative assay, the fragment with an unmethylated top strand and methylated bottom strand (lanes 23–28) achieved the same level of competition as the fully unmethylated fragment (lanes 3–8), even only at 6.25-fold excess. In contrast, the symmetrically methylated fragment (lanes 9–14) and the fragment with methylated top strand (lanes 17–22) could not eliminate the shift signal until at 50-fold excess. These results are reproducible (Supplementary Figure S2C) and suggest that top-strand methylation quantitively impacts YY1 binding to Tf_I motif 1 *in vitro*.

It is notable that the CpG dinucleotide is not fully conserved in genome-wide human or mouse YY1-binding motifs (Figure 1B). Even in some mouse L1 Tf monomer variants, C has been replaced by A or T, and G replaced by A (35). To explore the importance of either nucleotide in YY1 binding, we derived two mutant fragments and tested their ability to compete with a WT probe (Figure 2E). Both C > T (lanes 7–10) and G > A mutants (lanes 11–14) did not compete as well as the unlabeled WT fragment (lanes 3–6). Relative to the unlabeled WT fragment, the level of competition was at least 4 times weaker (compare mutants in 100-fold excess with WT in 25-fold excess), suggesting both nucleotides contribute to YY1 binding in the context of the Tf_I monomer.

Downstream to the central motif in the YY1-binding sequence are two well conserved thymidine residues (Figure 1B). To explore their roles in YY1 binding, we created unlabeled DNA fragments with one or both thymidines mutated (TT > AA, TT > AT, TT > TA or TT > TG) (Supplementary Figure S2D). In EMSA, none of these mutant fragments could effectively compete with WT fragments for YY1 binding (Supplementary Figure S2E). Among them, TT > AT performed the best: at 200-fold excess, it was able to eliminate the shift signal (lane 11), comparable to the WT fragment at 12.5-fold excess (lane 3). These results indicate that the two thymidines also play an important role in Tf_I 5′UTR’s interaction with YY1.

### An upstream pseudo motif in Tf_I monomers does not interact with YY1 protein

In addition to motif 1 that was predicted by earlier studies, we noted the presence of another string of nucleotides at nt 6–17 (CCGGCCACCTTC) that closely resembles the consensus YY1-binding site (Figure 1A). This motif is conserved at four out of the five core nucleotides (GCCAC instead of GCCAT). As tested below, it is unable to interact with YY1 protein, so we named it ‘pseudo motif’. To determine whether the pseudo motif interacts with YY1 protein we synthesized two unlabeled 30 bp DNA fragments for EMSA: a wild-type DNA fragment based on Tf_I M2, with the pseudo motif centrally located (Supplementary Figure S3A, WT2 GCCAC), and a mutant version with a single nucleotide substitution that restores it to the consensus YY1-binding site at the fifth core nucleotide position (Supplementary Figure S3A, Mut4 GCCAt). For this EMSA trial, because we were uncertain whether a biotin-labeled pseudo motif fragment would bind to YY1 protein and yield a shift signal, we decided to keep the biotin-labeled motif 1 fragment as the probe. In this design, binding of a DNA fragment to YY1 was evaluated by its potency as an unlabeled competitor. As a control (Supplementary Figure S3C, lanes 3–5), unlabeled motif 1 fragment diminished the shift in a quantitative manner: a significant reduction even at a 12-fold excess of unlabeled competitors and a 50-fold excess nearly eliminated probe binding. In contrast, the wild-type pseudo motif fragment could hardly compete with probe binding (Supplementary Figure S3C, lanes 6–8): some level of competition could only be discerned at 200-fold excess. Interestingly, unlabeled Mut4 fragment was able to reduce the shift in a dose-dependent manner although not as potent as the unlabeled motif 1 fragment (Supplementary Figure S3C, lanes 9–11). Similar results were obtained when nuclear protein extract from NIH/3T3 cells was used (Supplementary Figure S3D). These results suggest that the pseudo motif is unable to interact with YY1 protein due to its single nucleotide deviation from the consensus 5-nt core motif.

So far, we have used 30 bp DNA fragments as probes or competitors in EMSA trials. To evaluate the effect of sequence flanking the binding motif on YY1 protein binding, we progressively shortened the DNA fragments from the 3′ end, creating 26, 24 and 21 bp fragments (Supplementary Figure S3B; WT26, WT24 and WT21). When used as unlabeled competitors, the 26 and 24 bp fragments were similarly effective as compared to the 30 bp WT fragment (Supplementary Figure S3C, lanes 12 and 13). On the other hand, the 21 bp fragment was much less effective at 200-fold excess and only achieved the level of inhibition equivalent to the 30 bp WT fragment at 12-fold excess (Supplementary Figure S3C, compare lanes 3 and 14). These results suggest that the extra nucleotides flanking the YY1-binding motif potentiate its interaction with YY1 protein *in vitro*. Similar results were observed when the experiment was repeated with nuclear protein extract from NIH/3T3 cells (Supplementary Figure S3D).

### Knockdown of YY1 protein by siRNA reduces Tf_I promoter activity

**Figure 3.**
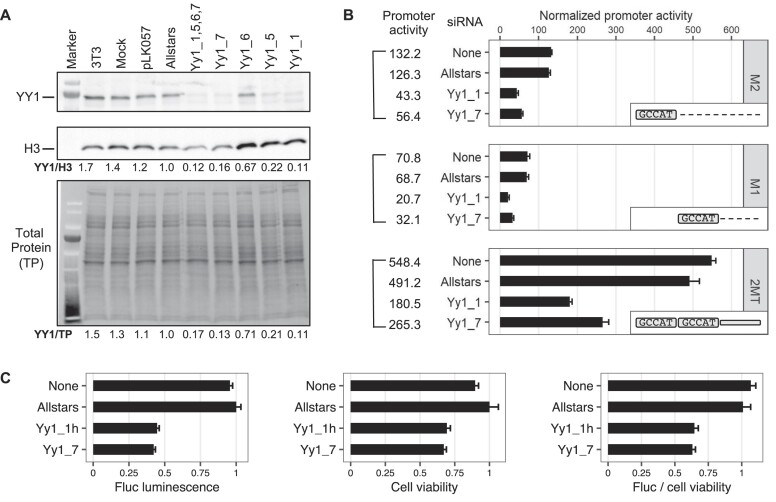
Knockdown of YY1 protein and its impact on the Tf_I promoter activity. (**A**) Western blot analysis of YY1 protein knockdown. NIH/3T3 cells were transfected with four YY1-specific siRNAs individually or as a pool. After 72 h whole cell lysates were probed for YY1 protein (top panel). Yy1_1 and Yy1_7 showed efficient knockdown compared to the control siRNA (Allstars). The YY1 protein signal was normalized to either histone H3 (middle panel) or fluorescent stain-free total protein signal (lower panel). (**B**) Normalized promoter activity for three Tf_I promoter constructs (M2, M1 and M2–M1–T) under siRNA knockdown. For each promoter variant, cells were cotransfected with the promoter construct and with or without a siRNA (y-axis; none = no siRNA). In reference to cells treated with Allstars, Yy1_1 siRNA treated cells showed 34.3%, 30.1% and 36.7% of the activity for M2, M1 and M2–M1–T, respectively. In comparison, Yy1_7 treated cells showed 44.7%, 46.7% and 54.0% of the activity for M2, M1 and M2–M1–T (marked as 2MT), respectively. The positive control construct, pCH117, had a normalized promoter activity of 1222.8. Error bars represent standard errors of the mean (*n* = 4). The inset illustrates the promoter constructs used. (**C**) Mouse L1 Tf_I promoter activity from a chromosomally integrated reporter under siRNA knockdown. A stable HCT116 cell line carrying an integrated mouse L1 Tf_I 5′UTR-Fluc reporter transgene was transfected with or without a siRNA (y-axis; none = no siRNA). In reference to cells treated with Allstars, Yy1_1h and Yy1_7 siRNA treated cells showed 44.7% and 42.3% in Fluc activity, 69.3% and 67.1% in cell viability (via CellTiter Blue assays), and 64.6% and 63.1% in normalized promoter activity (i.e. ratio of Fluc over cell viability), respectively. Error bars represent standard errors of the mean (*n* = 4).

So far, we have provided evidence that Tf_I 5′UTR loses its promoter activity when motif 1 is mutated (Figure 1) and motif 1 mediates the interaction between YY1 protein and Tf_I monomers (Figure 2). If YY1 protein binding to motif 1 is responsible for Tf_I promoter activity, a decrease in YY1 protein abundance should lead to reduced transcriptional activation. To knockdown YY1 protein, we first evaluated four siRNAs against mouse Yy1 RNA (Yy1_1, Yy1_5, Yy1_6 and Yy1_7) in NIH/3T3 cells by western blot (Figure 3A). Note the protein has an apparent molecular weight of 68 kD despite having a calculated molecular weight of 44.7 kD (17). As compared to a control non-specific siRNA, the highest knockdown efficiency was achieved by Yy1_1 (89%), followed by Yy1_7 (84%), Yy1_5 (78%) and Yy1_6 (33%). A knockdown efficiency of 88% was achieved when all four siRNAs were pooled together. Subsequently, we selected siRNA Yy1_1 and Yy1_7 for three Tf_I promoter assays (M2, M1 or M2–M1–T) in NIH/3T3 cells (Figure 3B). For each promoter group, its normalized promoter activity was largely unaffected by the negative control non-specific siRNA (Figure 3B; compare Allstars with no siRNA). In reference to Allstars, Yy1_1 siRNA treated cells showed 34.3%, 30.1% and 36.7% of the activity for M2, M1 and M2–M1–T, respectively. In comparison, Yy1_7 treated cells showed 44.7%, 46.7% and 54.0% of the activity for M2, M1 and M2–M1–T, respectively. When repeated in F9 cells, Yy1_1 siRNA treatment reduced M2, M1 and M2–M1–T activities to 46.3%, 60.7% and 43.5%, respectively; Yy1_7 siRNA decreased their activities to 56.1%, 71.4% and 51.7%, respectively (Supplementary Figure S4). These results confirm that YY1 functions as a transcriptional activator for Tf_I monomers in an episomal cell-based reporter assay.

**Figure 4.**
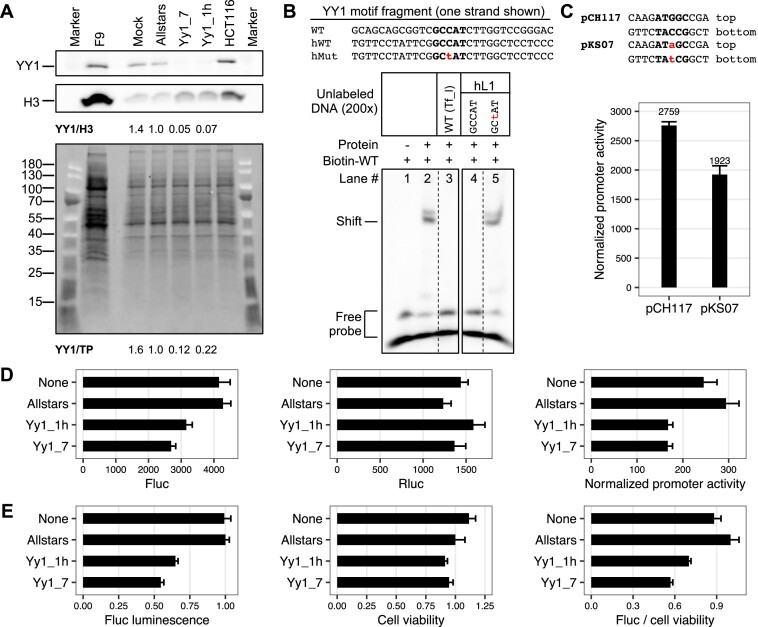
Effect of YY1 on human L1 promoter activity. (**A**) Western blot analysis of YY1 protein knockdown. HCT116 cells were transfected with individual YY1-specific siRNAs. After 72 h whole cell lysates were probed for YY1 protein (top panel). Yy1_1h and Yy1_7 showed efficient knockdown compared to the control siRNA (Allstars). The YY1 protein signal was normalized to either histone H3 (middle panel) or fluorescent stain-free total protein signal (lower panel). F9 cells were used as a control. (**B**) Competition EMSA using wild-type and mutant human L1 promoter fragments. Each fragment was formed by annealing a sense stranded oligo (shown) with the corresponding antisense oligo (not shown). Mutation in the core binding motif is indicated by lowercase in red. The presence or absence of a biotin-labeled Tf_I WT probe and nuclear protein extract from F9 cells is indicated by ‘+’ or ‘−’ symbols. Lanes 3–5 had unlabeled DNA fragments as competitors in 200-fold molar excess as indicated. A shift was observed in the presence of nuclear protein extract (lane 2). The shift was diminished by unlabeled WT Tf_I fragment (lane 3) and WT human L1 promoter fragment (hWT, lane 4) but not by unlabeled mutant human L1 promoter fragment (hMut, lane 5). All five lanes were from the same gel but they were separated by other unrelated samples; for clarity, lanes 1–3 and 4–5 were juxtaposed here. (**C**) Normalized promoter activity for wild-type and mutant human L1 5′UTR-Fluc reporters. Cells were transfected with either pCH117 or pKS07. The negative control construct, pLK037 is set to 1. Error bars represent standard errors of the mean (*n* = 4). (**D**) Promoter activity for human L1 5′UTR-Fluc reporter under siRNA knockdown. Cells were cotransfected with the reporter construct pCH117 and with or without an siRNA (y-axis; none = no siRNA). In reference to cells treated with the negative control siRNA (Allstars), Yy1_1h and Yy1_7 siRNA treated cells showed 73.8% and 63.0% in Fluc luminescence signal (left panel), respectively, and 57.0% and 56.8% in normalized promoter activity (right panel; the negative control construct, pLK037 is set to 1), respectively, after normalizing against Rluc luminescence (middle panel). In this assay, pCH117 without siRNA had a normalized promoter activity of 246. Error bars represent standard errors of the mean (*n* = 4). (**E**) Human L1 promoter activity from a chromosomally integrated reporter under siRNA knockdown. A stable HCT116 cell line carrying an integrated human L1 5′UTR-Fluc reporter transgene was transfected with or without an siRNA (y-axis; none = no siRNA). In reference to cells treated with the negative control siRNA (Allstars), Yy1_1h and Yy1_7 siRNA treated cells showed 64.9% and 54.6% in Fluc activity, 91.3% and 94.7% in cell viability, and 70.1% and 56.9% in normalized promoter activity (i.e. ratio of Fluc over cell viability), respectively. Error bars represent standard errors of the mean (*n* = 4).

To investigate the role of YY1 on Tf_I promoters within the chromosomal environment, we established a stable cell line that carries an integrated mouse L1 5′UTR-Fluc transgene. The mouse L1 5′UTR promoter is derived from L1spa (30,31), which is considered a prototypic mouse Tf_I element (34). Due to sequence and functional conservation between human and mouse YY1 proteins, we chose wild-type HCT116, a human colorectal cancer cell line, as the host. We targeted human YY1 transcripts with two siRNAs: Yy1_1h (human YY1-specific) and Yy1_7 (targeting a region homologous between mouse and human transcripts). As compared to a control non-specific siRNA, Yy1_1h and Yy1_7 achieved 93% and 95% knockout efficiency, respectively (Figure 4A). In reference to the negative control siRNA Allstars, Yy1_1h and Yy1_7 treated cells showed 44.7% and 42.3% in Fluc activity, respectively (Figure 3C, left panel). We detected a reduction in cell viability upon YY1 knockdown (Figure 3C, middle panel). After normalizing Fluc signals against cell viability, Yy1_1h and Yy1_7 treated cells showed 64.6% and 63.1% in normalized promoter activity, respectively (Figure 3C, right panel). These results indicate that YY1 transactivates mouse L1 Tf_I promoter in a chromosomal context.

### Effect of YY1 knockdown on human L1 promoter in transient and stable reporter assays

Human L1 5′UTR has a functional YY1-binding motif (25,26). To compare YY1’s role on human L1 with that on mouse L1 Tf_I promoter, we first checked whether the human L1 YY1-binding motif could compete with the mouse L1 YY1-binding motif in EMSA (Figure 4B). As expected, the shift signal was eliminated by both unlabeled human and mouse L1 fragments at 200-fold excess (lanes 3–4). As a control, a single nucleotide substitution in the core motif of human L1 fragment failed to compete with the biotin-labeled mouse L1 probe (lane 5). In episomal reporter assays, the corresponding mutant L1 promoter showed 30% reduction in promoter activity relative to the wild-type (Figure 4C). Moreover, the wild-type human L1 promoter activity was reduced to 57.0% and 56.8% of the control when HeLa cells were treated with Yy1_1h and Yy1_7 siRNAs, respectively (Figure 4D). In a stable HCT116 cell line carrying an integrated human L1 5′UTR-Fluc reporter (41), Yy1_1h and Yy1_7 treatment led to 64.9% and 54.6% in Fluc activity, respectively (Figure 4E, left panel). After normalizing Fluc signals against cell viability (middle panel), Yy1_1h and Yy1_7 treated cells showed 70.1% and 56.9% in normalized promoter activity, respectively (right panel). These experiments indicate that YY1 transactivates human L1 promoter in both episomal and chromosomal assays albeit to a lesser extent than mouse Tf_I promoter.

### Gf_I monomers lack a functional YY1-binding motif

**Figure 5.**
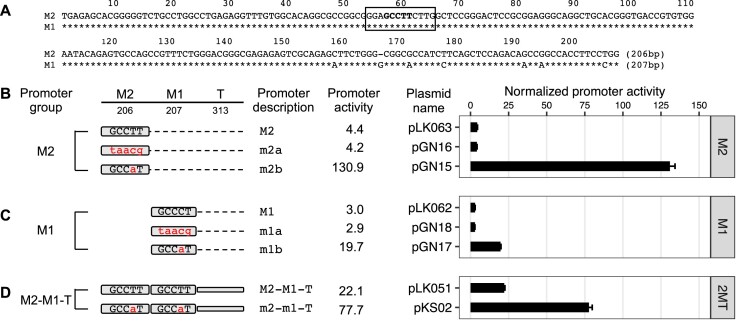
Promoter activities of YY1 motif variants of the Gf_I subfamily in F9 cells. (**A**) Alignment of Gf_I M2 and M1 consensus sequences. In the M1 sequence, nucleotide positions identical to M2 are marked by asterisks. Sequence gaps are represented by dashes. A previously predicted YY1-binding motif is located between nt 54–65 (solid box, termed ‘Gf_I motif’). Promoter activity is assessed using dual-luciferase reporter assay. (**B**) Normalized promoter activity of M2 constructs. Mutation to Gf_I motif (GCCCT) is indicated by lowercases in red. The m2a showed minimal change in promoter activity. However, changing to the consensus YY1 motif (m2b) elevated the M2 promoter activity by 29.8-fold. (**C**) Normalized promoter activity of M1 constructs. The mutant monomer 1 (m1a) showed minimal change in promoter activity. Changing to the consensus (m1b) showed 6.6 times higher signal compared to M1. (**D**) Normalized promoter activity of monomer2–monomer1–tether (M2–M1–T or 2MT) constructs. A 3.5-fold higher activity was observed upon changing both Gf_I motifs to the consensus sequence. The positive control construct, pCH117, had a normalized promoter activity of 493.0. Error bars represent standard errors of the mean (*n* = 4).

The Gf subfamily of mouse L1s was first reported in 2001 (36). The consensus Gf monomer shares sequence homology with Tf monomers. At the position corresponding to Tf_I motif 1, Gf monomer contains sequence GGAGCCTTCTTG, which deviates from the 5-nt consensus YY1-binding motif by one nucleotide (GCCTT instead of GCCAT) (Figure 5A). To determine whether this motif is important for Gf_I promoter activity, we created and tested two mutant variants for both Gf_I M2 and M1 in reporter assays in F9 cells (Figure 5B and C). The first mutant variant had all five core nucleotides altered (m2a and m1a, ‘taacg’). The same alteration completely abolished the promoter activity of Tf_I monomers in earlier experiments. However, minimal change in promoter activity was observed in the context of both Gf_I monomers (Figure 5B and C; compare m2a with M2 and compare m1a with M1), suggesting the original GCCTT motif in Gf_I monomers is not involved in transcriptional activation or repression. In the second mutant variant, we introduced a nucleotide substitution that effectively converted the original motif into a consensus YY1 core motif (m2b and m1b, ‘GCCaT’). Interestingly, this single nucleotide change elevated the Gf_I M2 promoter activity by 29.8-fold (Figure 5B, compare m2b with M2), even more active than the Tf_I M2 fragment (see Figure 1C). The same nucleotide change enhanced the M1 activity by 6.6-fold (Figure 5C, compare m1b with M1). Lastly, we introduced this nucleotide change to both monomers in the context of M2–M1–T and observed a significant 3.5-fold boost to the promoter activity (Figure 5D). Similar results were obtained from reporter assays conducted in NIH/3T3 cells (Supplementary Figure S5). Variant m2b, m1b and m2–m1–T showed 36.1-fold, 7.0-fold and 6.1-fold higher activities, respectively, than their wild-type counterparts. These data support the conclusion that the single nucleotide divergence in Gf_I monomers negatively affects its transcriptional output.

To explore whether these functional changes are mediated by YY1 transcription factor, we tested the ability of the wild-type and corrected Gf_I motif to interact with YY1 protein using EMSA. We synthesized two 30 bp DNA fragments for EMSA: a wild-type DNA fragment based on Gf_I M2, with the putative motif centrally located (Supplementary Figure S6A, WT GCCTT), and a mutant version with a single nucleotide substitution at the fourth core nucleotide position that restores it to the consensus YY1-binding site (Supplementary Figure S6A, Mut GCCaT). Indeed, when incubated with nuclear protein extract from F9 cells, a shift signal was absent for the biotin-labeled WT fragment (lane 2) but was present for the biotin-labeled Mut fragment (lane 4; Supplementary Figure S6B). To further evaluate differential binding by WT and Mut Gf_I monomer fragments, we set up a competition EMSA using the Mut probe (Supplementary Figure S6C). Again, nuclear protein extract bound to the GCCaT probe, causing a shift (lane 2). A supershift was observed with the addition of YY1 antibody (lane 3) but not with the addition of non-specific mouse IgG (lane 4), confirming the involvement of YY1 protein. The shift was diminished by an excess of unlabeled GCCaT DNA (lanes 5–7) but not by the WT DNA fragment (lanes 8–10). The inability of WT DNA fragments to compete for YY1 binding indicates that the single nucleotide difference prevents its interaction with YY1 protein. Similar results were obtained when nuclear protein extract from NIH/3T3 cells was used (Supplementary Figure S6D). Together, these results suggest that the homologous region in Gf_I monomers is unable to interact with YY1 protein due to its single nucleotide deviation from the consensus 5-nt core motif.

### YY1 activates Tf_I and Tf_II transcription during early mouse development

**Figure 6.**
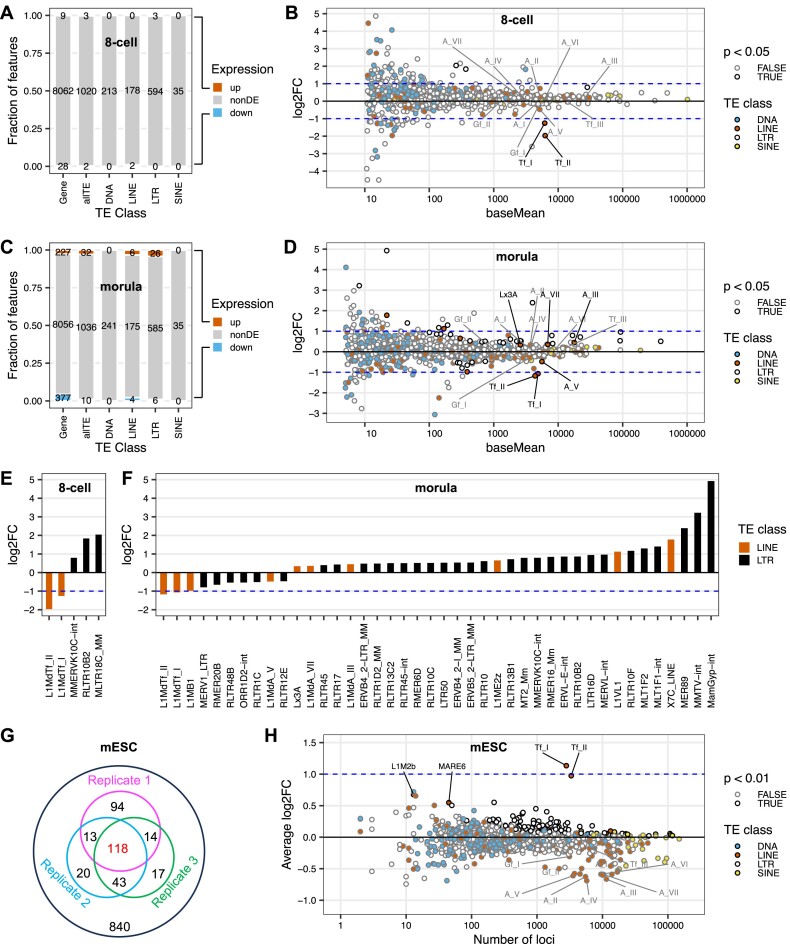
Zygotic knockdown of YY1 reduces Tf_I and Tf_II transcription in early mouse embryos. (**A–F**) Differential expression of genes and TEs in eight-cell embryos or morulas upon zygotic YY1 knockdown. Data were from Sakamoto *et al.* (38,43) and reanalyzed with TEtranscripts using repeat library db20140131. The proportions of upregulated (up), non-differentially expressed (non-DE) and downregulated (down) genes or TE subfamilies in eight-cell embryos (**A**) or morulas (**C**) are color-coded and plotted as stacked bar charts; the corresponding numbers of genes or TE subfamilies are marked. TEs are shown together (allTE) or as individual classes. log2FC of all TE subfamilies in eight-cell embryos (**B**) or morulas (**D**) are shown in MA plots. The four TE classes are color-coded as filled dots. TE subfamilies that display a statistically significant change in transcription (*P* < 0.05) are outlined in black. All statistically significant L1 subfamilies (black line and font; *P* < 0.05) as well as any remaining A, Gf and Tf subfamilies (gray line and font; *P* > 0.05) are labeled. Bar graphs list all statistically significant TE subfamilies in eight-cell embryos (**E**) or morulas (**F**). Note panels (B) and (D) use abbreviated subfamily names (e.g. Tf_I) while panels (E) and (F) display full names (e.g. L1MdTf_I). (**G, H**) Enrichment of YY1-binding among TE subfamilies in mouse embryonic stem cells (mESCs). Data were from Cusack *et al.* (45) and reanalyzed with T3E using repeat library db20140131. In panel G, each inner circle corresponds to the number of TE subfamilies enriched for YY1-binding in one of the three replicates (*P* < 0.01). (**H**) shows enrichment analysis of YY1-binding across 1159 TE subfamilies. The log2FC (y-axis) for each subfamily is plotted against its number of loci in the mouse genome. All statistically significant L1 subfamilies (black line and font; *P* < 0.01) as well as any remaining A, Gf and Tf subfamilies (gray line and font; *P* > 0.01) are labeled. Note the top ranked LTR subfamily RLTR43A (with log2FC of 2.9 and 3 genomic loci) is not shown on the graph.

Thus far, our investigation of YY1’s role as a transcriptional activator of mouse L1 has been limited to cell lines *in vitro*. In addition, we have relied on reporter assays due to inherent challenges in measuring endogenous L1 RNA (55). *In vivo*, L1 is most active during early embryogenesis, gametogenesis, neurogenesis and/or tumorigenesis [reviewed in (5)]. In particular, data from mouse models have established that early embryogenesis is a critical window of opportunity for L1 retrotransposition (56–60). During mouse development, L1 transcription peaks in two-cell embryos, declines in eight-cell embryos and further decreases in 16-cell embryos (61,62). In parallel, YY1 protein is first detected in two-cell embryos and maintained at high levels beyond 16-cell morula-stage embryos (43). The timing of L1 and YY1 expression during early mouse embryogenesis suggests a potential involvement of YY1 in transcriptional activation of mouse L1 Tf subfamilies *in vivo*. Indeed, Sakamoto and colleagues recently demonstrated reduced L1 expression in morulae upon zygotic YY1 knockdown (38). Specifically, their differential expression analysis in morulae showed > 50% reduction for both L1Md_Gf and L1Md_T subfamilies after YY1 knockdown. Such results are inconsistent with a prediction based on our *in vitro* data, which clearly showed transcriptional activation of Tf_I but not Gf_I promoters. We reasoned that this discrepancy could potentially be caused by different TE annotations used by us and by the previous study. The default TE annotation for the mouse genome assembly GRCm38/mm10 is based on RepeatMasker library release 20110920, which classifies mouse L1 subfamilies into L1Md_A, L1Md_Gf, L1Md_T, L1Md_F/F2/F3. In contrast, our study is based on the updated mouse L1 nomenclature as defined by Sookdeo and colleagues (27), in which the old L1Md_A subfamily corresponds to A_I, A_II and A_III, the old L1Md_T subfamily corresponds to Tf_I, Tf_II, Tf_III, Gf_II and part of Gf_I, the old L1Md_F subfamily corresponds to part of Gf_I, Fanc_I and N_I, the old L1Md_F2 subfamily corresponds to A_IV, A_VII and F_I to F_V and the old L1Md_F3 subfamily corresponds to A_V, A_VI and part of N_I. We thus reanalyzed YY1 knockdown data reported previously (38,43) (Supplementary Table S4) using RepeatMasker library db20140131, which incorporated the updated mouse L1 nomenclature, and via the TEtranscripts package (44) (Figure 6; Supplementary Table S5). At the eight-cell stage, only a small number of genes are differentially expressed as a result of zygotic YY1 knockdown (Figure 6A), including 9 upregulated genes and 28 downregulated genes. Similarly, only five TE subfamilies showed differential expression at this stage. Specifically, three upregulated TE subfamilies all belong to LTR class, and two downregulated TE subfamilies are both L1 subfamilies (Tf_I and Tf_II) (Figure 6E). Tf_I reduced to 41.8% of the control and Tf_II reduced to 25.5% of the control. Importantly, no other evolutionally young L1 subfamilies showed statistically significant changes in transcription at the eight-cell stage (Figure 6B). In morula embryos, 227 genes are upregulated and 377 genes are downregulated. Meanwhile, 32 TE subfamilies are upregulated (26 LTR and 6 LINE) and 10 TE subfamilies downregulated (6 LTR and 4 LINE) (Figure 6C). Again, Tf_I and Tf_II subfamilies remain downregulated (at 47.7% and 44.3% of the control, respectively) (Figure 6F). Most of the evolutionarily young L1 subfamilies showed no statistically significant changes in transcription, with the exception of A_V, A_III and A_VII (Figure 6D and F). It is important to note that we reproduced results reported in the original publication when we switched to the default TE annotation file (Supplementary Figure S7). Thus, Tf_I and Tf_II subfamilies but not Gf_I or Gf_II subfamilies are transcriptionally activated during early mouse development.

A prediction from the analysis above is that, among different mouse L1 subfamilies, YY1 would preferentially bind to Tf_I and Tf_II elements during early embryogenesis. To compare YY1 occupancy among TE subfamilies we utilized Transposable Element Enrichment Estimator (T3E), a recently published ChIP-seq analysis pipeline specifically designed to profile protein binding across TE families/subfamilies (46). T3E computes the number of read mappings for a TE subfamily by counting both unique- and multiple-mapped reads. The degree of enrichment is reflected by FC, a ratio between read mappings in the ChIP-seq sample and the average of simulations based on the input library. Thus, for this analysis we selected a previously published YY1 ChIP-seq dataset from mESCs with an input library (45) (Supplementary Table S4). Among 1159 TE subfamilies encompassing LINE, SINE, LTR retrotransposons and DNA transposons, 10% (118/1159) were found enriched for YY1 binding (*P* < 0.01) across all three ChIP-seq samples (Figure 6G; Supplementary Table S6). Only three TE subfamilies displayed two-fold or higher FCs. RLTR43A, a member of LTR/ERVK family with only three genomic loci, has the highest average FC (7.5) among the three ChIP-seq samples. The second- and third-ranked subfamilies are Tf_I and Tf_II (2.2 and 2.0, respectively) (Figure 6H). In total, 4 L1 subfamilies showed enrichment for YY1 (Figure 6H). However, none of the other evolutionarily young mouse L1 subfamilies were enriched for YY1 binding, such as Tf_III, Gf_I, A_I, A_II and N_I (Figure 6H).

## Discussion

Unlike human L1, the role of YY1 in mouse L1 transcription had not been experimentally tested before. In this study, we examined YY1’s function in the transcription of two mouse L1 subfamilies, known as Tf and Gf when they were initially discovered (30,36). We provide multiple lines of evidence that support an activating role of YY1 for Tf subfamilies: (i) In luciferase-based reporter assays, mutating the conserved nucleotides of the putative YY1-binding site diminished the promoter activity in four different promoter constructs (M2 alone, M1 alone, M1–T and M2–M1–T) (Figure 1). (ii) In EMSA, 30 bp DNA fragments containing the putative YY1-binding motif showed sequence-specific interaction with YY1-containing nuclear protein extract (Figure 2). (iii) siRNA knockdown of YY1 protein led to reduced promoter activities in both episomal and chromosomally integrated reporter assays (Figure 3). In parallel, we provided experimental evidence that excluded a role of YY1 for Gf subfamily: (i) In reporter assays, mutating all five core nucleotides in the putative YY1-binding motif had no effect on activity of M2 or M1. In contrast, a single nucleotide substitution restoring to the consensus led to multi-folds of increase in promoter activity for three promoter constructs (M2, M1 and M2–M1–T) (Figure 5). (ii) In EMSA, while DNA fragments containing the single nucleotide substitution interacted with YY1-containing nuclear protein extract, DNA fragments containing the putative YY1-binding motif failed when used as a competitor (Supplementary Figure S6). These data indicate that the lack of YY1 binding is the result of a single nucleotide deviation in Gf promoter (GCCTT) from the consensus YY1-binding motif (GCCAT). Additionally, we excluded the presence of a second YY1-interacting motif in Tf monomers. This motif (the pseudo motif) bears a GCCAC core sequence, also a single nucleotide deviation from the YY1 consensus (GCCAT). We showed that (i) In EMSA, DNA fragments containing the wild-type GCCAC motif could not compete for YY1 binding. DNA fragments containing a single nucleotide substitution restoring it to the consensus had enhanced interaction with YY1-containing nuclear protein extract although not as efficient as the motif 1 containing DNA (Supplementary Figure S3). (ii) In reporter assays, when motif 1 (GCCAT) was mutated to GCCAC the activity of M2 was diminished (Figure 1), suggesting GCCAC is not compatible for YY1 binding.

Importantly, our observation helps to explain the synergy between Tf monomers. We previously dissected the relative contribution of M2, M1 and the tether sequence to the overall promoter activity for A_I, Gf_I and Tf_I subfamilies (34). For A_I subfamily, M2 is the major contributor, M1 has minimal activity and the tether negatively regulates M2 in the context of two-monomer 5′UTR. The Gf_I subfamily has the lowest promoter activity tested, with contribution from a synergistic interaction between M2 and the tether. For Tf_I subfamily, it appears that M2 and M1 are synergistic while the tether is additive to the overall two-monomer promoter activity. However, trans-acting factors mediating the synergistic interaction between Tf_I M2 and M1 were unknown. In the current study, we showed that an intact YY1 motif was required for the activity of each monomer when tested on its own and in the context of two tandem monomers and that simultaneously mutating both YY1 motifs eliminated the two-monomer promoter activity (Figure 1 and Supplementary Figure S1). YY1 can form dimers and high-order oligomers (63). YY1 dimerization promotes interactions between enhancers and promoters (21,23). Thus, it is conceivable that tandem arrayed Tf monomers may be bridged together via YY1 binding and multimerization. Under this model, distant monomers may act like enhancers and synergistically boost the activity of the proximal monomers (Supplementary Figure S8). By positioning multiple YY1-binding monomers immediately upstream, Tf_I and Tf_II 5′UTRs are configured like a housekeeping gene promoter, which tends to have built-in enhancer activities mediated by GABPA or YY1 motifs (64). As a result, it may help to minimize the influence of distal enhancers on L1 promoter activity. This model is consistent with a recent report of methylation patterns across the entire mouse L1 5′UTR in undifferentiated mESCs: the inner monomers are consistently hypomethylated in elements containing three or more monomer units (60). A similar mechanism likely operates at other genes with clustered YY1-binding sites, such as the X-inactive specific transcript (Xist) highlighted below (65,66).

Heritable insertions occur during early embryogenesis and/or gametogenesis (40,58,59). An *in vivo* regulatory role of YY1 for Tf (but not Gf) subfamily expression during early embryogenesis is strongly supported by our secondary analyses of published RNA-seq data in early-stage mouse embryos (38,43) and ChIP-seq data in mESCs (45). Specifically, upon zygotic knockdown of YY1, Tf_I and Tf_II are the only two TE subfamilies showing reduced RNA levels in eight-cell embryos, and this downregulation persists in morula-stage embryos (Figure 6A–F). We recommend the use of an updated genomic TE annotation (RepeatMasker library db20140131) when classifying mouse L1 subfamilies. Indeed, when the default TE annotation table (RepeatMasker library release 20110920 for GRCm38/mm10) was used, we reproduced the findings in the original publication, which reported a downregulation of Gf expression upon zygotic YY1 knockdown (38) (Supplementary Figure S7). Consistent with RNA-seq results in embryos, our secondary analysis of YY1 ChIP-seq data in mESCs indicates that Tf_I and Tf_II are the only two evolutionarily young mouse L1 subfamilies with high enrichment of YY1 binding (Figure 6G and H). Unlike Tf_I and Tf_II monomers, Tf_III monomers are degenerate at the underlined nucleotide position in the YY1-binding site (GTCGCCATCTKG; K = T or G). This nucleotide position is highly conserved among YY1-binding sites (Figure 1B). According to our EMSA data (Supplementary Figure S2E), the deviation from the consensus (T > G) leads to reduced YY1 interaction and helps to explain why Tf_III subfamily is not enriched for YY1 binding in the ChIP-seq data. Thus, our findings that YY1 is a transcriptional activator of Tf subfamilies sheds light on their high retrotransposition activity relative to other evolutionarily young mouse L1 subfamilies (59,67).

Our study of YY1’s role in mouse L1 regulation was proceeded by pioneer investigations of YY1’s function for human L1s (25,26). The prevailing model for YY1’s function in human L1 is that YY1 mainly controls the precision of transcriptional initiation but has minimal impact on the overall transcriptional output from the full-length 5′UTR promoter (26). To directly compare YY1’s transactivating role between human and mouse L1s, we conducted parallel experiments using *in vitro* binding assays as well as episomal and chromosomal reporter assays. We confirmed that an intact binding motif in human L1 5′UTR is critical for its interaction with YY1-containing nuclear protein extract (Figure 4B) as previously reported (25,26). However, a copy of human L1 5′UTR carrying the exact nucleotide substitution in the YY1-binding motif only showed 30% reduction in its promoter activity when transiently transfected into HeLa cells (Figure 4C). This result was in sharp contrast to what we observed for mouse Tf_I promoter (for example, a minimum of 74% reduction was seen even when only one monomer was mutated; see Figure 1E and Supplementary Figure S1C for F9 and NIH/3T3 cells, respectively). On the other hand, this result was consistent with ∼50% reduced activities previously observed in two human embryonal carcinoma cell lines although the same study did not find a reduction in promoter activity in HeLa cells (26). Cell subline differences could potentially explain the discrepancy between this and previous study using HeLa cells. In any case, it should be noted that a minimal human L1 promoter encompassing the first 150 bps have been shown to be much more sensitive to mutations in the YY1-binding motif, showing 66% to 94% reduction in three cell lines, including HeLa, when the binding motif was either scrambled or deleted (25,26). We further demonstrated 30% to 45% reduction of human L1 promoter activity in both episomal and chromosomal reporter assays upon siRNA knockdown of YY1 (Figure 4D and E). Together these results suggest that YY1 also transactivates a full-length human L1 but perhaps to a lesser degree than a mouse L1 Tf promoter.

In 1997, Austen and colleagues proposed that the ubiquitously expressed YY1 functions as ‘a permanently present basal transcription factor whose activity is controlled by secondary events’ (19). It has now been established that DNA methylation is such a secondary event. The inability of YY1 to bind to methylated motifs has been demonstrated for mouse intracisternal A-type particle retrotransposon (52,53), imprinting control regions of multiple imprinted genes (e.g. Nespas, Peg3, Tsix and Xist) (54,65,66,68) and, most recently, across the genome (69). In this regard, Xist exemplifies methylation-dependent regulation of YY1 function. Xist is a long non-coding RNA that serves as the master regulator of X-chromosome inactivation. The presence of clustered YY1-binding sites is evolutionarily conserved in mammalian Xist promoters (65). In both human and mouse female cells, YY1 binds solely to the unmethylated Xist allele and activates its expression in an allele-specific manner (65,66,68). The YY1-binding motif in mouse L1 Tf monomers (GTCGCCATCTTG) is highly similar to the long high-affinity YY1-binding motifs found in Peg3 and Xist (GCCGCCATTTTG) (70). Using EMSA, we found that replacing the cytosine with 5-methylcytosine at the CpG dinucleotide position (underlined above) reduced YY1’s binding affinity to Tf_I promoter fragments by at least 8-fold (Figure 2). Interestingly, this interference of YY1 binding by DNA methylation is mediated solely by the 5-methylcytosine on the top strand (Figure 2D). Our results mirror previous EMSA data for one of the YY1-binding motifs in the first intron of Peg3 (GGCGCCATCTTT) (54). The strand-specific effect could potentially be explained by the asymmetric binding of YY1 to its binding motifs as revealed by X-ray crystallography and *in vitro* binding experiments (70,71). Taken together, we expect YY1 to behave similarly at Tf promoters: binding and activating unmethylated motifs but unable to bind and function when Tf monomers are methylated.

The large number of YY1-binding sites in TEs has implications for gene regulation at the genome level. A total of 118 subfamilies showed varied enrichment of YY1 occupancy in all three ChIP-seq libraries from mESCs (Figure 6G), including 99 subfamilies from the LTR class, 11 subfamilies from the SINE class, 7 subfamilies from the LINE class and one subfamily from the DNA transposon class (Supplementary Table S6). Although enriched to less extent at individual subfamily level than Tf_I and Tf_II, together these subfamilies provide a formidable collection of YY1-binding sites. There is a striking parallel in the human genome. YY1-binding sites have been identified in all four classes of human retrotransposons. For LTR element, a YY1-binding site is found in the U3 region of HERV-K and activates HERV-K transcription (72). For the SINE class, a YY1-binding site is in the left monomer of Alu, downstream from the RNA Pol III promoter (73–75). A DNA fragment containing this binding motif interacts with recombinant YY1 protein *in vitro* although showing lesser affinity when compared to a fragment containing the canonical YY1-binding site (74). Lastly, a composite YY1-OCT4 binding motif or a YY1 motif alone is enriched in SVAs that are transcribed in human induced pluripotent stem cells (76). Thus, in both mouse and human genomes, YY1-binding motifs in TEs may contribute to the enhancer-promoter interaction network (21). Consistent to this hypothesis, full-length Tf elements display hypomethylated monomers (60) and increased chromatin accessibility (77) in mESCs. Indeed, a recent study demonstrates that a subset of Tf 5′UTRs function as enhancer to regulate naive pluripotency in mESCs (78).

Our study has several limitations. First, our results provide insights into transcriptional regulation of the mouse L1 Tf subfamily but not into how Gf and A subfamilies are regulated, both of which lack functional YY1-binding sites. Second, our work focuses on YY1’s role on transcriptional activation. Whether YY1 regulates transcription initiation of Tf monomers remains to be investigated. Third, the current work only measured promoter activities. The impact of YY1 on Tf element retrotransposition should be tested in the context of the whole element in a retrotransposition assay in the future. Fourth, our reanalysis of published data supports YY1’s role as a transcriptional activator during early embryonic development. Future studies should examine whether YY1 functions as a transcriptional activator in other developmental time points.

## Supplementary Material

gkae949_Supplemental_Files

## Data Availability

All data generated or analyzed during this study are either plotted or detailed in supplemental information.
