# Supplementary material for: YY1 is a transcriptional activator of the mouse LINE-1 Tf subfamily
Source: Nucleic Acids Res. 2024 Oct 26;52(21):12878–94. doi: 10.1093/nar/gkae949 (PMC11602158; doi:10.1093/nar/gkae949)
Supplement: gkae949_Supplemental_Files [file gkae949_supplemental_files.zip › YY1-figures-suppl-202409.pdf]

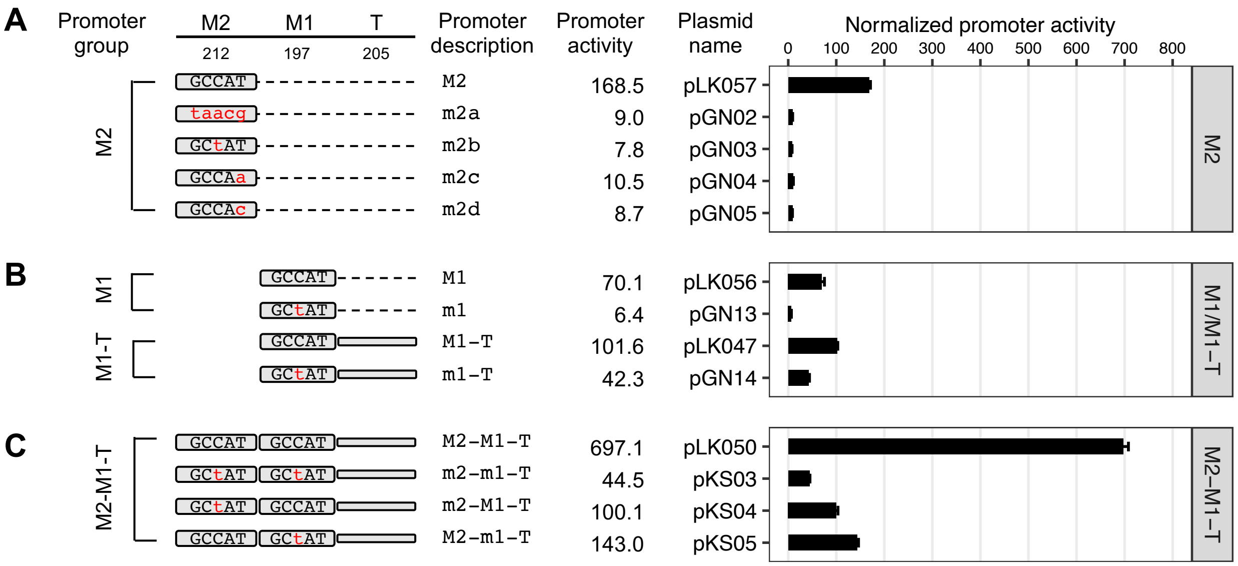

Figure S1. Effect of YY1 motif mutants on Tf_I promoter activity in NIH/3T3 cells. (**A**) Normalized promoter activity of M2 constructs. Mutation to consensus YY1 binding motif 1 (GCCAT) is indicated by lowercases in red. The four mutant promoter variants uniformly displayed minimal promoter activity, corresponding to 16- to 22-fold reduction as compared to the wild-type. (**B**) Normalized promoter activity of M1 and M1-Tether (M1-T) constructs. Mutant M1 (m1) showed 11-fold less activity than M1. A 2.4-fold reduction was seen in m1-T as compared to M1-T. (**C**) Normalized promoter activity of M2-M1-T constructs. The promoter activity was reduced by 16-fold when both monomers were mutated. Mutation to one monomer at a time showed activity from the other monomer and tether. For panels A-C, sequence organization of the promoters is illustrated on the left side. The length of M2, M1, and tether for each promoter is annotated (in base pairs). The dashed line represents domain(s) that were removed in reference to the two-monomer 5'UTR sequence (M2-M1-T). The x-axis indicates the normalized promoter activity, which is also listed under column “promoter activity” for each promoter variant. The positive control construct, pCH117, had a normalized promoter activity of 1526.3. Error bars represent standard errors of the mean (n = 4).

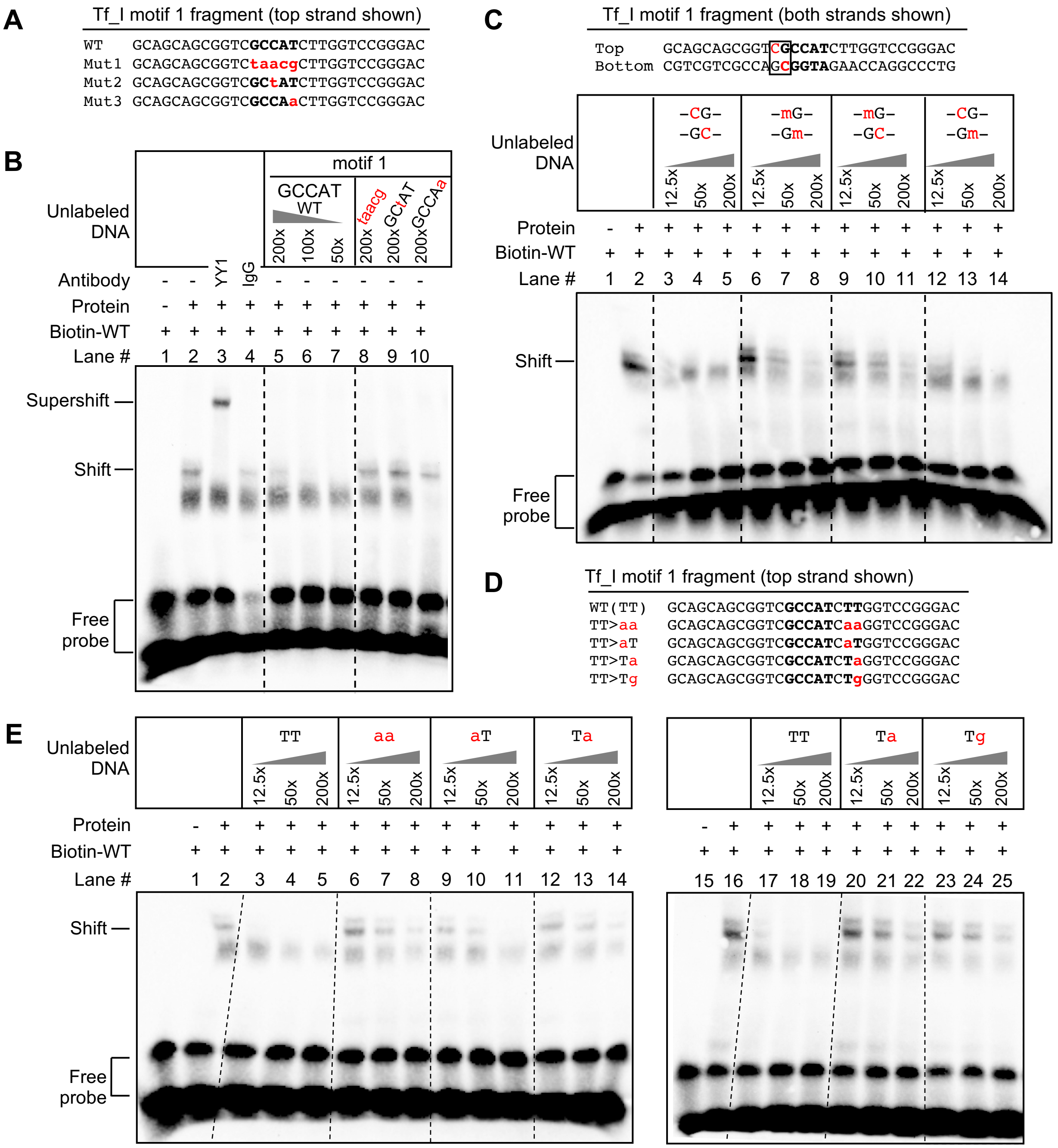

Figure S2. Interaction of YY1 protein with motif 1. (**A**) Wild-type and mutant DNA fragments used in electrophoretic mobility shift assays (EMSA). Each fragment was formed by annealing a sense stranded oligo (shown) with the corresponding antisense oligo (not shown). Mutations in the core binding motif are indicated by lowercases in red. (**B**) EMSA with Tf_I motif 1 fragments. The presence or absence of a biotin-labeled WT probe, antibody, and nuclear protein extract from NIH/3T3 cells is indicated by “+” or “-” symbols. Lanes 5-10 had unlabeled DNA fragments as competitors in molar excess as indicated. A shift was observed in the presence of nuclear protein extract. A supershift was observed with the addition of YY1 antibody (lane 3) but not by mouse IgG (lane 4). The shift was diminished by increasing amount of unlabeled WT fragment but not by unlabeled mutant DNA fragments (Mut1, Mut2, and Mut3). (**C**) EMSA using unmethylated and variably methylated Tf_I motif 1 DNA fragments as competitor. The complete sequence of the unmethylated fragment (double stranded) is shown at the top with the CpG dinucleotide boxed. A biotin-labeled unmethylated WT probe is used. Competitors used for specific lanes are indicated by the CpG position (m, 5-methylcytosine). Note this is a repetition of experiment in Fig.2D but with a single gel. The presence or absence of a biotin-labeled WT probe and nuclear protein extract from F9 cells is indicated by “+” or “-” symbols. (**D**) Wild-type and mutant DNA fragments used in EMSA. Each fragment was formed by annealing a sense stranded oligo (shown) with the corresponding antisense oligo (not shown). Mutations in two thymidine residues are indicated by lowercases in red. (**E**) EMSA with Tf_I motif fragments containing mutations in the two thymidine positions. The presence or absence of a biotin-labeled WT probe and nuclear protein extract from NIH/3T3 cells is indicated by “+” or “-” symbols. Two gels are used to accommodate all reactions. Lanes 3-14 and 17-25 had unlabeled DNA fragments as competitors in molar excess as indicated.

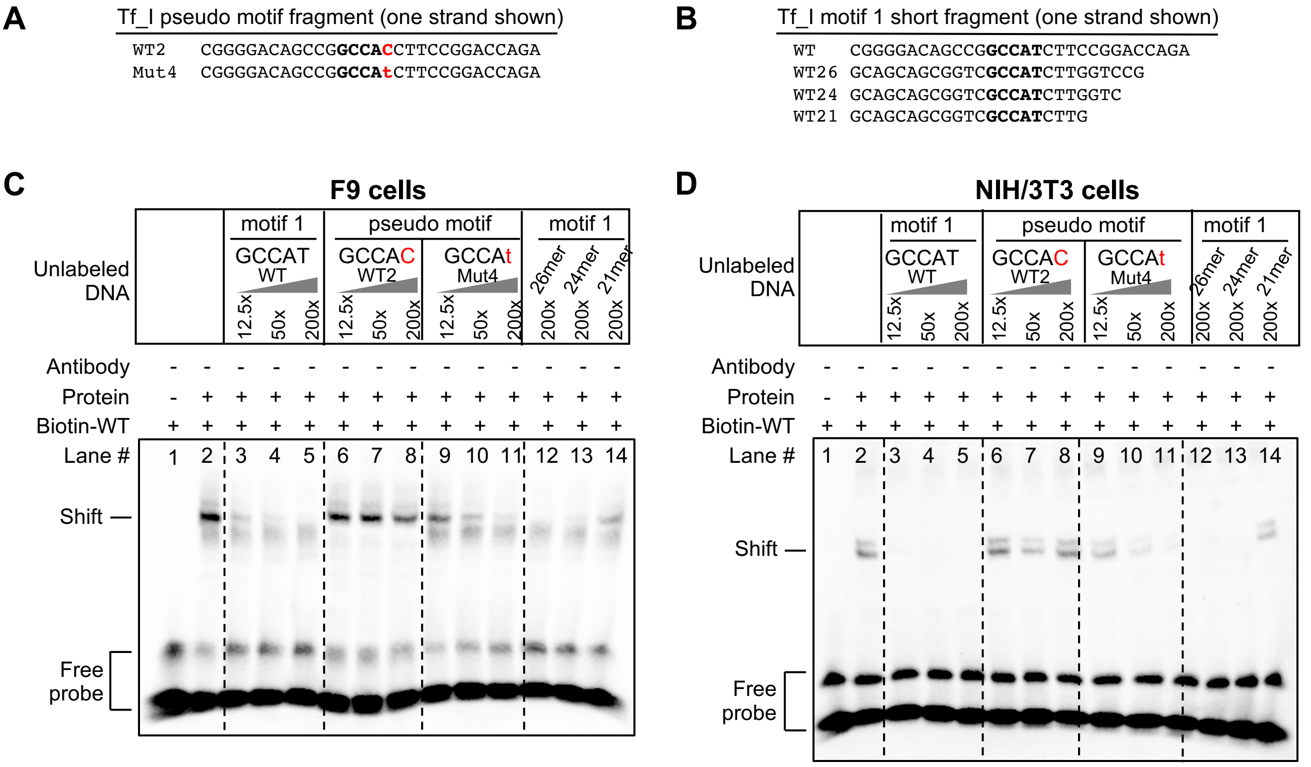

Figure S3. No interaction of YY1 protein with a pseudo motif in Tf_I monomers. (**A,B**) Wild-type and mutant DNA fragments used in electrophoretic mobility shift assays (EMSA). Each fragment was formed by annealing a sense stranded oligo (shown) with the corresponding antisense oligo (not shown). Mutations in the core binding motif are indicated by lowercases in red. The wild-type pseudo motif (WT2) differs from motif 1 (WT) at the fifth position of the core binding sequence. (**C,D**) EMSA with Tf_I pseudo motif fragments and shortened motif 1 fragments. The presence or absence of a biotin-labeled WT probe, antibody, and nuclear protein extract is indicated by "+" or "-" symbols. Lanes 3-14 had unlabeled DNA fragments as competitors in molar excess as indicated. The shift was diminished by an unlabeled mutant fragment containing the consensus core motif (Mut4; lanes 9-11) by not by unlabeled wild-type pseudo motif fragment (WT2; lanes 6-8). Shortened motif 1 containing DNA fragments (26bp or 24bp) were able to inhibit the shift but 21bp fragments were not as effective (lane 14). Panels C and D used nuclear protein extracts from F9 cells and NIH/3T3 cells, respectively.

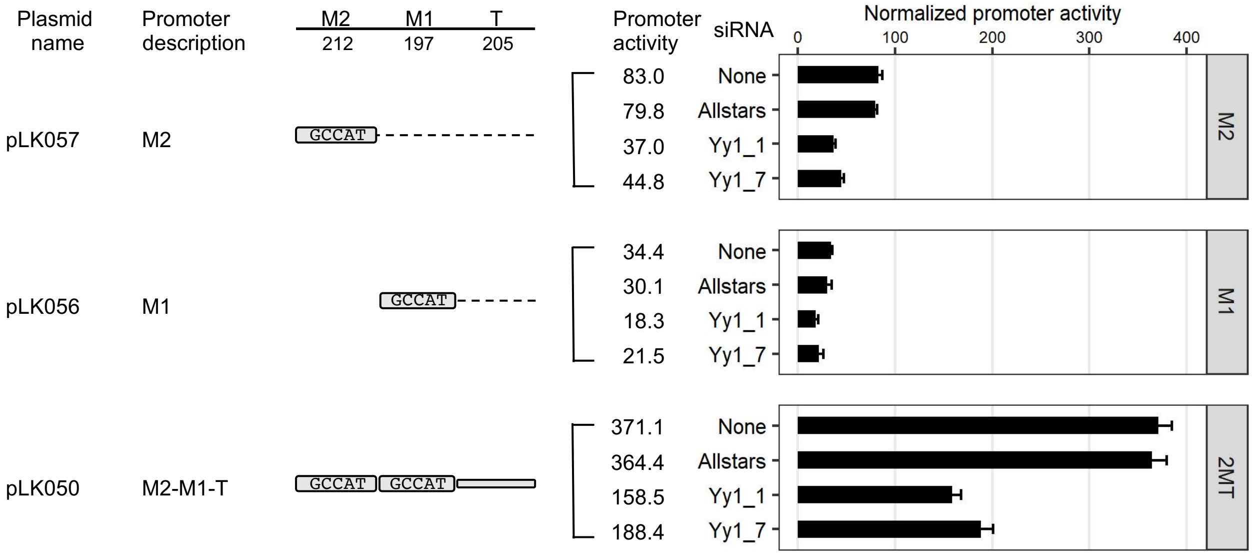

Figure S4. Knockdown of YY1 protein and its impact on the Tf_I promoter activity in F9 cells. Normalized promoter activity for three Tf_I promoter constructs (M2, M1, and M2-M1-T) under siRNA knockdown. For each promoter variant, cells were cotransfected with the promoter construct and with or without a siRNA (y axis; none = no siRNA). In reference to cells treated with Allstars, Yy1_1 siRNA treated cells showed 46.3%, 60.7%, and 43.5% of the activity for M2, M1, and M2-M1-T, respectively. In comparison, Yy1_7 treated cells showed 56.1%, 71.4%, and 51.7% of the activity for M2, M1, and M2-M1-T (also marked as 2MT), respectively. The positive control construct, pCH117, had a normalized promoter activity of 950.2. Error bars represent standard errors of the mean (n = 4).

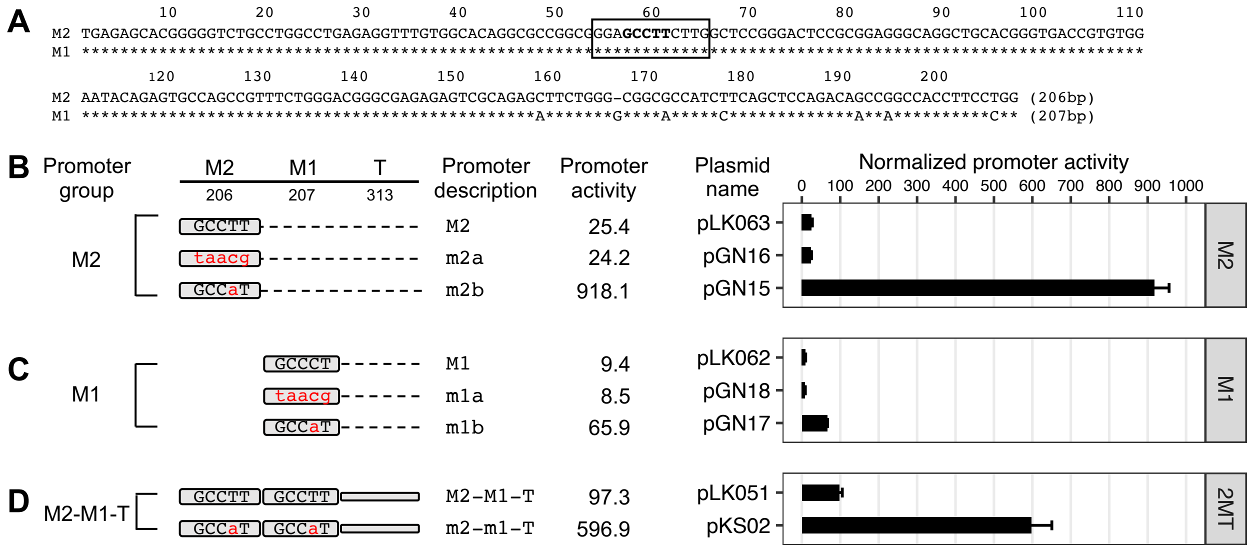

Figure S5. Promoter activities of YY1 motif variants of the Gf_I subfamily in NIH/3T3 cells. (**A**) Alignment of Gf_I monomer 2 (M2) and monomer 1 (M1) consensus sequences. In the M1 sequence, nucleotide positions identical to M2 are marked by asterisks. Sequence gaps are represented by dashes. A previously predicted YY1 binding motif is located between nts 53-64 (solid box, termed "Gf_I motif"). Promoter activity is assessed using Dual-luciferase reporter assay. **(B)** Normalized promoter activity of monomer2 (M2) constructs. Mutation to Gf_I motif (GCCCT) is indicated by lowercases in red. The m2a showed minimal change in promoter activity. However, changing to the consensus YY1 motif (m2b) elevated the M2 promoter activity by 36.1-fold. **(C)** Normalized promoter activity of monomer1 (M1) constructs. The mutant monomer 1 (m1a) showed minimal change in promoter activity. Changing to the consensus (m1b) showed 7.0 times higher signal compared to M1. **(D)** Normalized promoter activity of monomer2-monomer1-Tether (M2-M1-T; or 2MT) constructs. A 6.1-fold higher activity was observed upon changing both Gf_I motifs to the consensus sequence. The positive control construct, pCH117, had a normalized promoter activity of 1942.5. Error bars represent standard errors of the mean (n = 4).

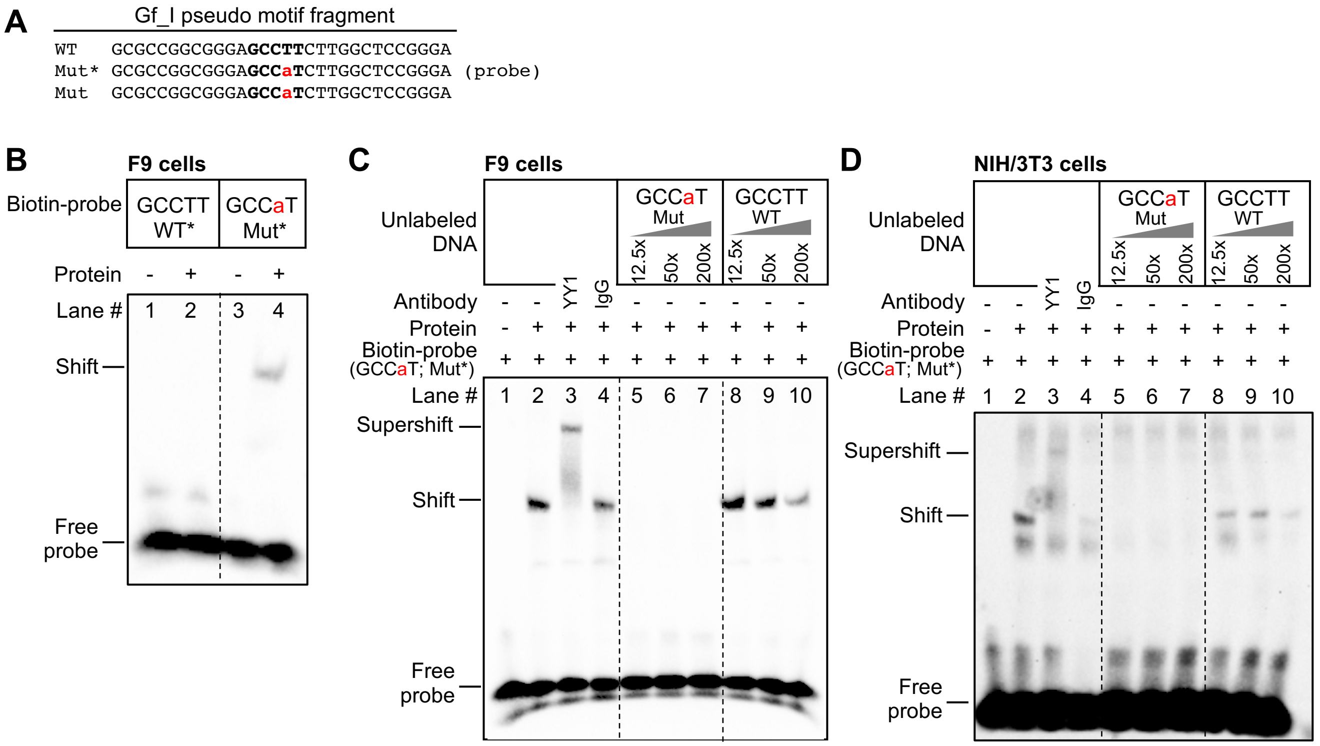

Figure S6. Lack of interaction of a putative YY1 binding motif in Gf_I monomers with nuclear protein extracts from F9 and NIH/3T3 cells. (**A**) Wild-type and mutant DNA fragments used in EMSA. Each fragment was formed by annealing a sense stranded oligo (shown) with the corresponding antisense oligo (not shown). Mutation in the core binding motif is indicated by a lowercase letter in red. In the mutant variant (Mut), the wild-type pseudo motif (WT) was mutated at the fourth position to restore the consensus core binding sequence. (**B**) EMSA using WT and Mut Gf_I pseudo motif fragments as probes. The presence or absence of nuclear protein extract from F9 cells is indicated by "+" or "-" symbols. Lanes 1-2 had biotin-labeled WT fragment. Lanes 3-4 had biotin-labeled Mut fragment. (**C**) Competitive EMSA using Gf_I pseudo motif fragments. The presence or absence of a biotin-labeled Mut probe (GCCaT), antibody, and nuclear protein extract from F9 cells is indicated by "+" or "-" symbols. Lane 3 had YY1-specific antibody and lane 4 had mouse IgG as a control. Lanes 5-10 had unlabeled DNA fragments as competitors in molar excess as indicated. (**D**) Competitive EMSA using Gf_I pseudo motif fragments. The presence or absence of a biotin-labeled Mut probe (GCCaT), antibody, and nuclear protein extract from NIH/3T3 cells is indicated by "+" or "-" symbols. Lane 3 had YY1-specific antibody and lane 4 had mouse IgG as a control. Lanes 5-10 had unlabeled DNA fragments as competitors in molar excess as indicated.

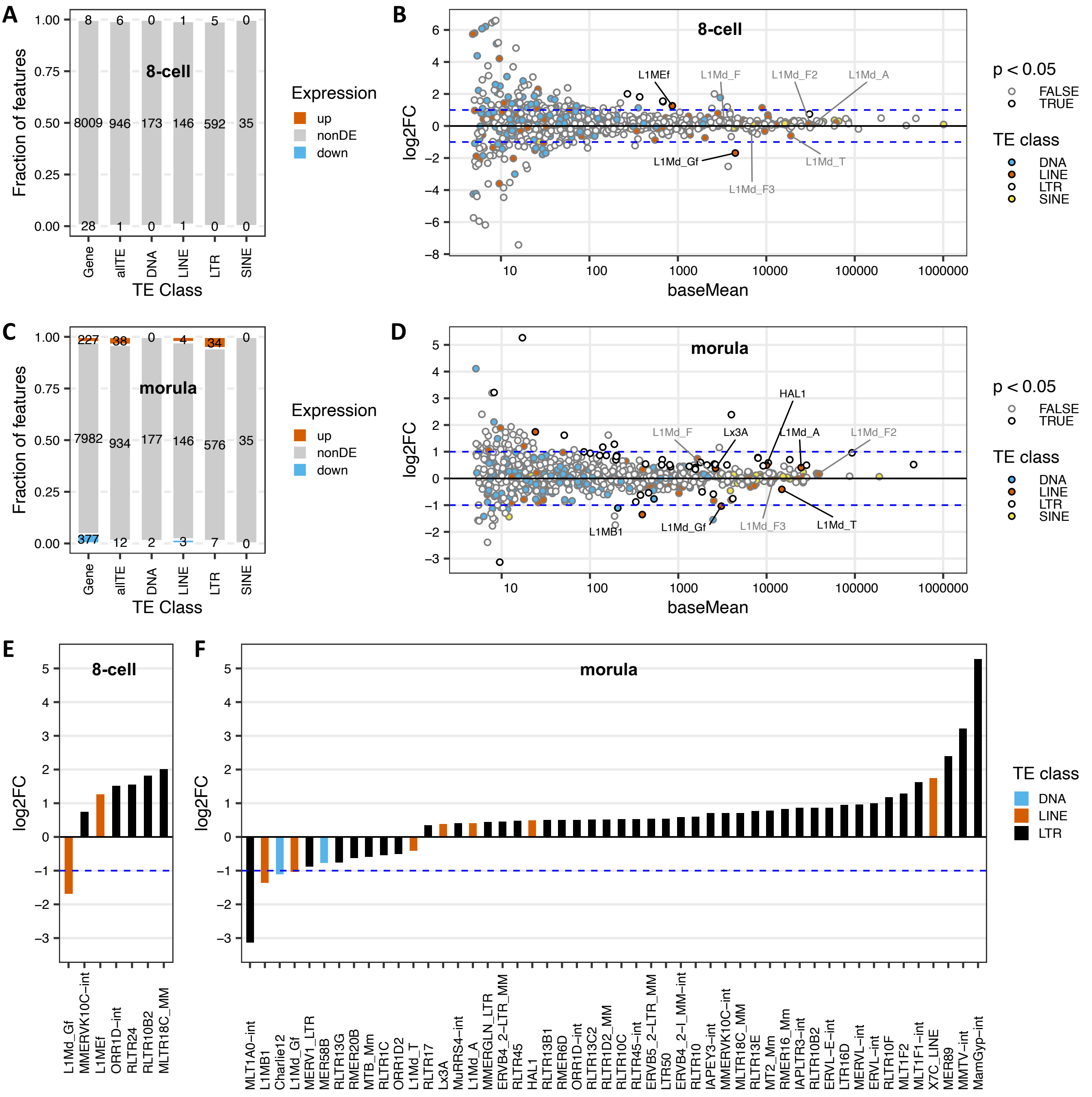

Figure S7. TEtranscripts analysis of Sakamoto's RNA-seq data using default repeat library. (**A-F**) Differential expression of genes and TEs in 8-cell embryos or morulas upon zygotic YY1 knockdown. Data were from Sakamoto et al and reanalyzed with TEtranscripts using default repeat library. The proportions of upregulated (up), non-differentially expressed (nonDE), and downregulated (down) genes or TE subfamilies in 8-cell embryos (**A**) or morulas (**C**) are color-coded and plotted as stacked bar charts; the corresponding numbers of genes or TE subfamilies are marked. TEs are shown together (allTE) or as individual classes. Log2 fold changes (log2FC) of all TE subfamilies in 8-cell embryos (**B**) or morulas (**D**) are shown in MA plots. The four TE classes are color-coded as filled dots. TE subfamilies that display a statistically significant change in transcription ($p < 0.05$) are outlined in black. All statistically significant L1 subfamilies (black line and font; $p < 0.05$) as well as any remaining A, Gf, T, and F/F2/F3 subfamilies (gray line and font; $p > 0.05$) are labelled. Bar graphs list all statistically significant TE subfamilies in 8-cell embryos (**E**) or morulas (**F**).

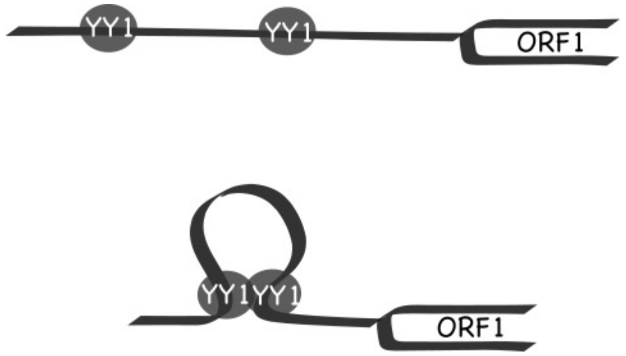

Figure S8. Model of YY1-mediated promoter synergy between Tf monomers. (Top) A two-monomer 5'UTR promoter sequence is positioned upstream of the ORF1 coding sequence. Each monomer harbors a functional YY1-binding site, which is occupied by a YY1 molecule. (Bottom) The two YY1 molecules dimerize and form a transcription hub, resulting in synergistic increase in promoter activity. In this model, the upstream monomer functions as an enhancer for the downstream monomer.
